# Genomic sequencing of *Thinopyrum elongatum* chromosome arm 7EL, carrying fusarium head blight resistance, and characterization of its impact on the transcriptome of the introgressed line CS-7EL

**DOI:** 10.1186/s12864-022-08433-8

**Published:** 2022-03-23

**Authors:** David Konkin, Ya-Chih Hsueh, Morgan Kirzinger, Marie Kubaláková, Aparna Haldar, Margaret Balcerzak, Fangpu Han, George Fedak, Jaroslav Doležel, Andrew Sharpe, Thérèse Ouellet

**Affiliations:** 1 Aquatic and Crop Resource Development, National Research Council of Canada, 110 Gymnasium Place, Saskatoon, SK S7N 0W9 Canada; 2 Institute of Experimental Botany of the Czech Academy of Sciences, Šlechtitelů 31, CZ-77900 Olomouc, Czech Republic; 3 Ottawa Research and Development Centre, Agriculture and Agri-Food Canada, 960 Carling Ave, Ottawa, ON K1A 0C6 Canada; 4 Department of Biology, University of Ottawa, Ottawa, ON K1N 6N5 Canada; 5 State Key Laboratory of Plant Cell and Chromosome Engineering, Institute of Genetics and Developmental Biology, Chinese Academy of Sciences No1, Beijing, China; 6 Global Institute for Food Security, 110 Gymnasium Place, Saskatoon, SK S7N 0W9 Canada

**Keywords:** *Fusarium graminearum*, Disease resistance, *Thinopyrum elongatum*, *Triticum aestivum*, Transcriptome, Alien introgression, RNA-seq, Non-coding RNA

## Abstract

**Background:**

The tall wheatgrass species *Thinopyrum elongatum* carries a strong fusarium head blight (FHB) resistance locus located on the long arm of chromosome 7 (7EL) as well as resistance to leaf and stem rusts, all diseases with a significant impact on wheat production. Towards understanding the contribution of *Th. elongatum* 7EL to improvement of disease resistance in wheat, the genomic sequence of the 7EL fragment present in the wheat Chinese Spring (CS) telosomic addition line CS-7EL was determined and the contribution and impact of 7EL on the rachis transcriptome during FHB infection was compared between CS and CS-7EL.

**Results:**

We assembled the *Th. elongatum* 7EL chromosome arm using a reference-guided approach. Combining this assembly with the available reference sequence for CS hexaploid wheat provided a reliable reference for interrogating the transcriptomic differences in response to infection conferred by the 7EL fragment. Comparison of the transcriptomes of rachis tissues from CS and CS-7EL showed expression of *Th. elongatum* transcripts as well as modulation of wheat transcript expression profiles in the CS-7EL line. Expression profiles at 4 days after infection with *Fusarium graminearum,* the causal agent of FHB, showed an increased in expression of genes associated with an effective defense response, in particular glucan endo-1,3-beta-glucosidases and chitinases, in the FHB-resistant line CS-7EL while there was a larger increase in differential expression for genes associated with the level of fungal infection in the FHB-susceptible line CS. One hundred and seven 7EL transcripts were expressed in the smallest 7EL region defined to carry FHB resistance.

**Conclusion:**

7EL contributed to CS-7EL transcriptome by direct expression and through alteration of wheat transcript profiles. FHB resistance in CS-7EL was associated with transcriptome changes suggesting a more effective defense response. A list of candidate genes for the FHB resistance locus on 7EL has been established.

**Supplementary Information:**

The online version contains supplementary material available at 10.1186/s12864-022-08433-8.

## Background

Fusarium head blight (FHB), caused by *Fusarium graminearum* Schwabe (*Hypocreales: Nectriaceae*) and closely related species, is an economically important disease in wheat, barley, oats and maize in all temperate regions of the world [1]. In hexaploid wheat (*Triticum aestivum* L.) FHB causes significant losses every year in many of the wheat producing countries [2–4]. In addition to quantitative losses from reduced yield, FHB causes qualitative damages associated with the production of mycotoxins, including deoxynivalenol and derivatives. Research efforts over the last few decades have identified wheat germplasm with resistance to FHB, including Sumai 3, Wangshuibai, Wuhan 1, Frontana and CM-82036 [5]. Although those sources of resistance are used frequently in wheat improvement programs, only wheat varieties with moderate resistance to FHB have been generated so far, in part due to the complex genetic make-up of the resistance mechanisms involved.

The wild grass species *Thinopyrum elongatum* (syn. *Lophopyrum elongatum*, *Agropyron elongatum*), commonly referred to as tall wheatgrass, has been identified as a source of strong resistance to FHB [6, 7]. Using wheat addition lines carrying single *Th. elongatum* chromosomes, FHB resistance has been mapped to the long arm of chromosome 7E (7EL) [7–9]. This resistance is of great interest because it protects wheat against FHB to a high level, and also because the source is located on a single chromosome arm, possibly at a single locus, in contrast to other sources of resistance to FHB that are complex and multi-genic. In addition, 7EL also carries the resistance genes Lr19 and Lr29 for leaf rust, and Sr25 and Sr43 for stem rust [10–12].

Molecular characterization of 7EL is required to advance towards identification of its genetic loci for FHB and rust resistance genes. This study presents the 7EL genomic sequence from the addition line CS -7EL, and incrementally a transcriptomic study focusing on the contribution of 7EL to FHB resistance in wheat rachis, including a list of the 7EL transcripts expressed from the region associated with FHB resistance.

## Results

### Genomic sequence of chromosome 7EL from *Th. elong*atum

Isolation of a long arm fragment of the 7E chromosome of *Th. elongatum* from meristem root-tip cells of the Chinese Spring telosomic addition line CS-7EL was performed using flow cytometry [13]. The considerably smaller size of the 7EL telocentric chromosome compared to the native wheat chromosomes (Additional file 1) enabled isolation of 7EL chromosomal fragments with high purity (94%). We assembled the 7EL chromosomal fragment using paired-end libraries with a range of input sizes (Additional file 2). We selected the Ray assembler [14] for initial assembly based on a comparison of assembler performance on amplified flow-sorted wheat chromosomes using Sanger-sequenced wheat bacterial artificial chromosome sequences as a reference (unpublished data). This resulted in a base assembly with a size consistent with the estimated size of the 7EL fragment (353 Mbp).

To augment the base assembly, we prepared multiple mate pair libraries derived from nuclear DNA of CS-7EL, with insert sizes ranging from 2.8 to 40 kbp (Additional file 2). These mate pairs were used to scaffold the 7EL sequence as well as the IWGSC Chinese Spring chromosomal draft sequence. Gap filling resulted in an assembly with a scaffold N50 length of 81.6 Kbp and a total length of 330 Mbp (Table 1). We refer to the 7EL assembly derived here as “Dvorak74” in reference to the original source of the 7EL telosomic addition line [15].

**Table 1:** Statistics for the Dvorak74 7EL genomic sequence assembly

Number of contigs	162,390
Total contig length	306 Mbp
Contig N50 length	10,065 bp
Number of scaffolds	23,512
Total scaffold length	330 Mbp
Scaffold N50 length	81,629 bp
Number of anchored scaffolds	7657
Total anchored scaffold length	280 Mbp
LTR transposons	241,151 (158 Mbp)
DNA transposons	213,510 (75 Mbp)
Rachis expressed gene models	2450
Genes models transferred from D-3458 assembly	6422

Comparison of BUSCO [16] assessment scores for our 7EL assembly with matched segments from chromosomes 7A, 7B and 7D of the wheat RefSeq v1.0 assembly [17] supported the completeness of gene space in the 7EL assembly (Additional file 3). The scaffolded and gapfilled Chinese Spring assembly was released as the IWGSC draft assembly v3 [18].

We next performed a reference-guided assembly of the 7EL scaffolds using an existing assembly (ASM1179987v1, referred to hereafter as D-3458) of the *Th. elongatum* accession D-3458 [19, 20]. This resulted in 280 Mbp of sequence anchored to the D-3458 assembly, of which 231 Mbp (83%) corresponded to chromosome 7 of the D-3458 assembly.

### Comparison of the Dvorak74 7EL assembly with the D-3458 reference

**Fig. 1:**
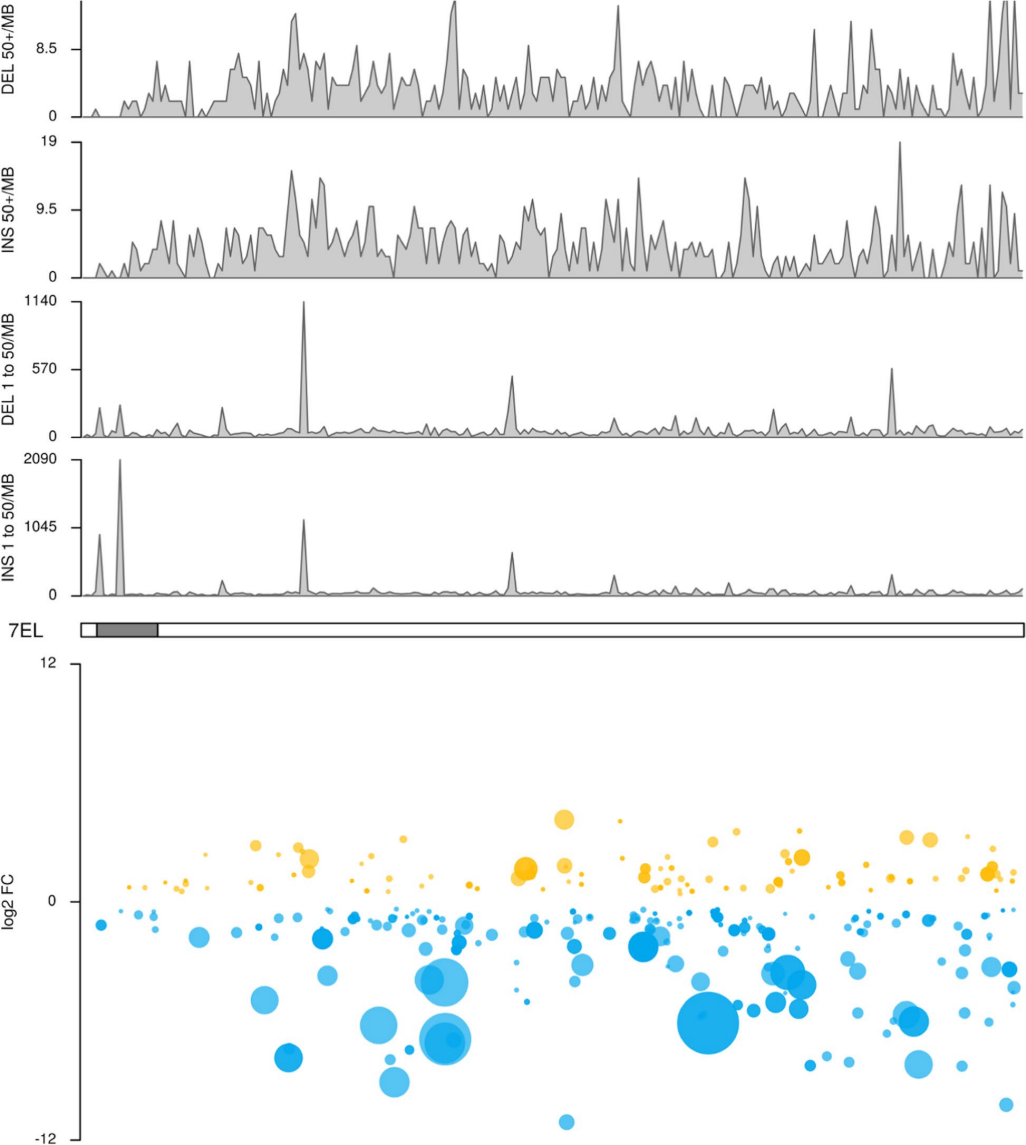
Dvorak74 7EL Karyoplots. Upper panel: Number of structural variants relative to the D-3458 assembly along the 7EL pseudomolecule. The estimated location of the centromere is represented as a gray box. Lower panel: Differentially expressed 7EL transcripts from CS-7EL rachis in response to *F. graminearum* infection; 7EL transcripts are represented as dots with color representing direction of change in expression, vertical position representing degree of difference in expression (expressed as log_2_ fold change), dot size representing statistical significance and horizontal position their location along the 7EL pseudomolecule

**Fig. 2:**
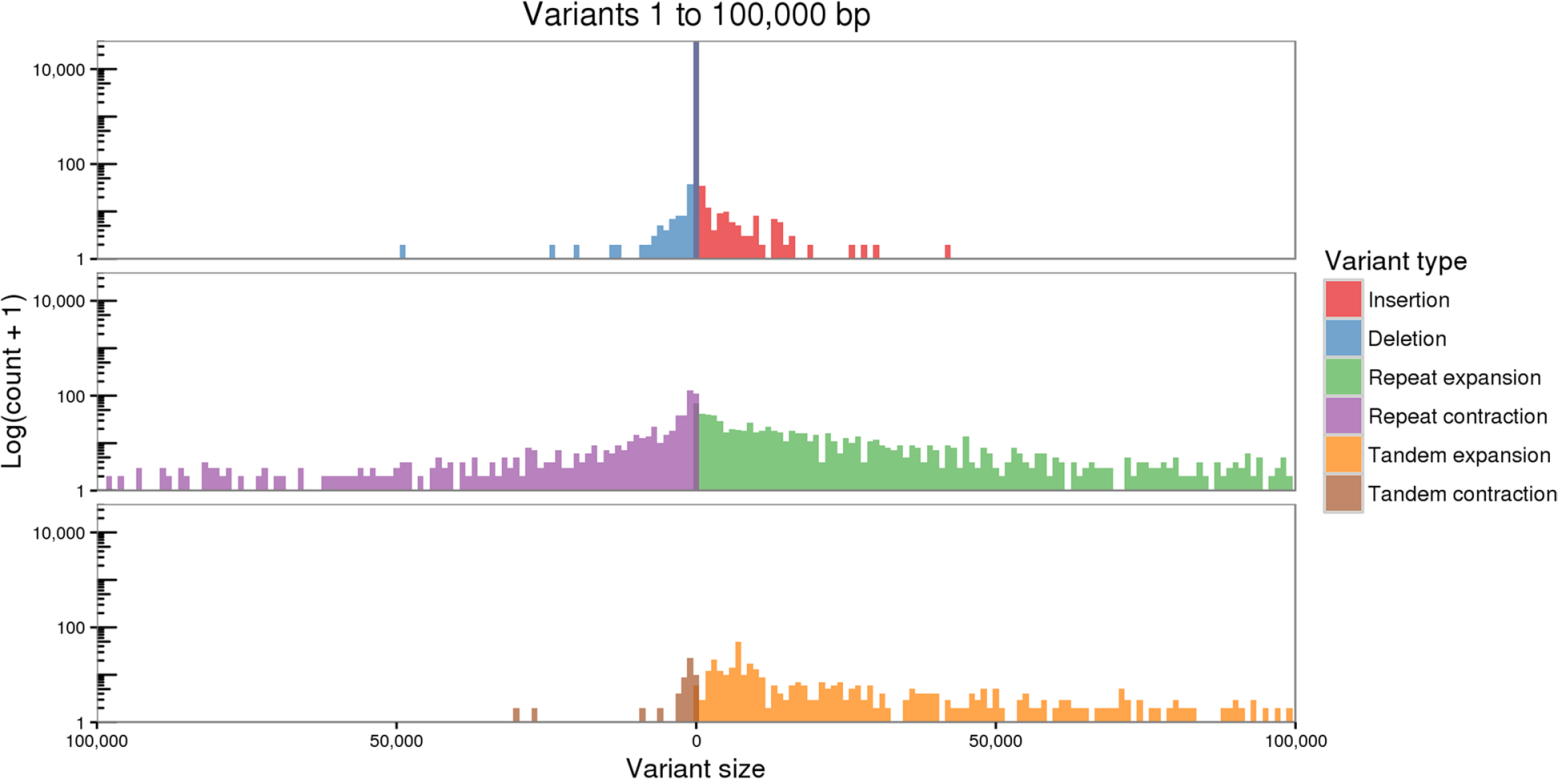
Distribution of structural variation between Dvorak74 and D-3458 assemblies of *Th. elongatum* chromosome arm 7EL. For each variant type, Y-axis shows the number of structural variants (expressed as log(count+ 1)) for each given size (X-axis)

Comparison of the 7EL assembly component with the D-3458 assembly identified a large number of differences (Figs. 1, 2, Additional file 4). Overall, 81,241 structural differences affecting 35.1 Mbp were identified, of which 2122 (34.94 Mbp) were structural variants of size greater than 50 bp. Four hotspots of small structural differences (50 bp or less) were identified (Fig. 1, lower panel), with two being localized to the centromeric region, as estimated by the localization of centromere specific repeats [20]. Larger structural variants were well distributed across the 7EL chromosome (Fig. 1, lower panel).

Classification of structural variants according to their context revealed that the majority of expansions and contractions occur in repetitive elements (Fig. 2), consistent with previous findings that transposable elements underlie a large proportion of structural variants in plant genomes [21]. There is an apparent bias toward tandem expansion in the Dvorak74 assembly (Fig. 2). We suspect that this bias reflects differences in assembly algorithms used for the two assemblies [19, 20], with the Ray assembler showing a greater propensity to represent as multiple copies what the DeNovoMagic assembler represents as a single copy. We therefore encourage downstream users to use additional caution when interpreting functional consequences of these putative structural variants in the tandem context.

We also identified 501,757 SNPs between the two assemblies in the 7EL region with a transition to transversion ratio of 2.08. An average nucleotide diversity of 0.0022 between the two assemblies compares closely to median values measured within the A genomes of diverse wild einkorn wheat and wild emmer wheat populations [22]. These polymorphisms strongly support the hypothesis that the introgression source of the 7EL in this study was distinct from the D-3458 accession used by Wang et al. [19].

Our flow-sorted Dvorak74 7EL chromosome assembly provided an opportunity to bin unanchored scaffolds from the D-3458 scaffolds to the 7EL region. While the D-3458 reference assembly is arranged in pseudomolecules, 96 Mbp of sequence divided in 639 scaffolds remain unplaced. Of the 639 unplaced scaffolds in the D-3458 reference assembly 115 have assignments to our 7EL scaffolds. We classified 46 of these scaffolds representing 13 Mbp of unanchored sequence as having strong support for localization to the 7EL region (Additional file 5).

Since our Dvorak74 assembly was built from flow-sorted chromosomes, Dvorak74 scaffolds mapping to other chromosomes could represent real structural differences, or alternatively may represent misplaced scaffolds or missing 7EL sequences in the D-3458 reference assembly. It is possible that the small amount of non-7EL DNA present in the 7EL flow sorted prep could have been assembled in the Dvorak74 assembly. The high purity of the 7EL fragments, as judged by fluorescence and the fairly even distribution of assignments of Dvorak74 7EL scaffolds to non-7EL chromosomes of the D-3458 assembly during reference guided scaffolding (Additional file 6) support the contrary.

### Assembly annotation

Like many other grass chromosomes, the 7EL assembly consists primarily of transposable elements, with LTRs being the dominant class (Table 1). Using rachis RNA-seq data described below, we built de novo 2540 gene models, of which 961 were placed in the chromosome level assembly. We also lifted over 6422 gene annotations from chromosome 7 of the D-3458 assembly [20]. Of these lifted over reference annotations, 623 had a match to the 961 transcripts (65%) that were identified de novo in this work.

We classified CS and 7EL transcripts according to their coding potential using two algorithms, CNIT and CPC2 [23, 24]. The two algorithms were largely congruent though CPC2 tended toward classifying transcripts as non-coding (Table 2, Additional file 7). Almost all (98%) of the discrepancies for 7EL transcripts were due to CPC2 classifying as non-coding and CNIT as coding. Discrepancies in classification for CS transcripts followed the same trend but was less pronounced with 68% of discrepancies being CPC2 non-coding and CNIT coding. Comparing the classifications of isoforms, roughly 5% of isoform for a given gene were classified differently by a single program and these transcripts tended to be sources of discrepancies between the two programs (Additional file 7). Subsequent comparisons of coding and non-coding transcripts are based on transcripts and genes where the two programs agreed. Overall, the ratio of non-coding to coding transcripts was substantially higher in 7EL (1:2.1) compared to CS (1:5.3) (Table 2).

**Table 2:** Predicted coding potential for 7EL and CS wheat genes

	7EL			wheat		
	CPC2	CNIT	Agreement	CPC2	CNIT	Agreement
**Coding**	1146	1767	1102	100,821	110,798	92,832
**Non-coding**	1264	642	627	45,173	36,051	30,131
**Mixed**	130	89	40	9898	8223	5184
**Not annotated**		42			820	
**Total annotated**	2540	2498	1769	155,892	155,072	128,147

### Contribution and impact of the 7EL chromosome arm on the transcriptome of the wheat rachis in CS background

To investigate the contribution and impact of the 7EL chromosome arm to gene expression in the CS background, we compared CS gene expression profiles between rachis tissues of CS and CS-7EL sampled at 4 days after treatment of spikelets with either water (control) or *F. graminearum*.

First, we examined the impact of the alien 7EL chromosome arm on wheat gene expression by comparing the abundance of the wheat transcripts in presence and absence of the 7EL chromosome arm using the control (water treated) samples. There were 282 wheat transcripts that were significantly (log2FC > |2|, padj< 0.001) differentially expressed (DE) between CS and CS-7EL, 174 of them being repressed and 108 upregulated in the presence of the 7EL chromosome arm (Additional file 8). These DE transcripts represented only 0.22% of all of the wheat transcripts expressed in the control samples.

The CS transcripts that were impacted by the presence of the 7EL chromosome arm under control conditions could be associated with a functional category represented by a broad range of functions. Putative lncRNAs were the most abundant, with 10% of the DE transcripts; however, about 59% of the DE transcripts were either annotated as uncharacterized proteins or had no homology in the databases searched (Additional file 9). The DE transcripts were distributed across all wheat chromosomes and subgenomes and were more frequently downregulated in CS-7EL, which is consistent with a dosage compensation model (Additional file 10). Two notable exceptions to that trend were observed on 6B and 7B where larger numbers of wheat transcripts were upregulated in the presence of the 7EL chromosome arm, with most of the DE transcripts from 7B (46/50) representing more than 66% of the upregulated transcripts with a log2FC > 5 (Additional file 8). Interestingly, all but one of the 7B wheat transcripts strongly upregulated in the presence of chromosome arm 7EL were located in a relatively narrow segment of the chromosome, between positions 383,502,275 and 397,828,549.

### Differences in response to FHB between CS and CS-7EL

**Fig. 3:**
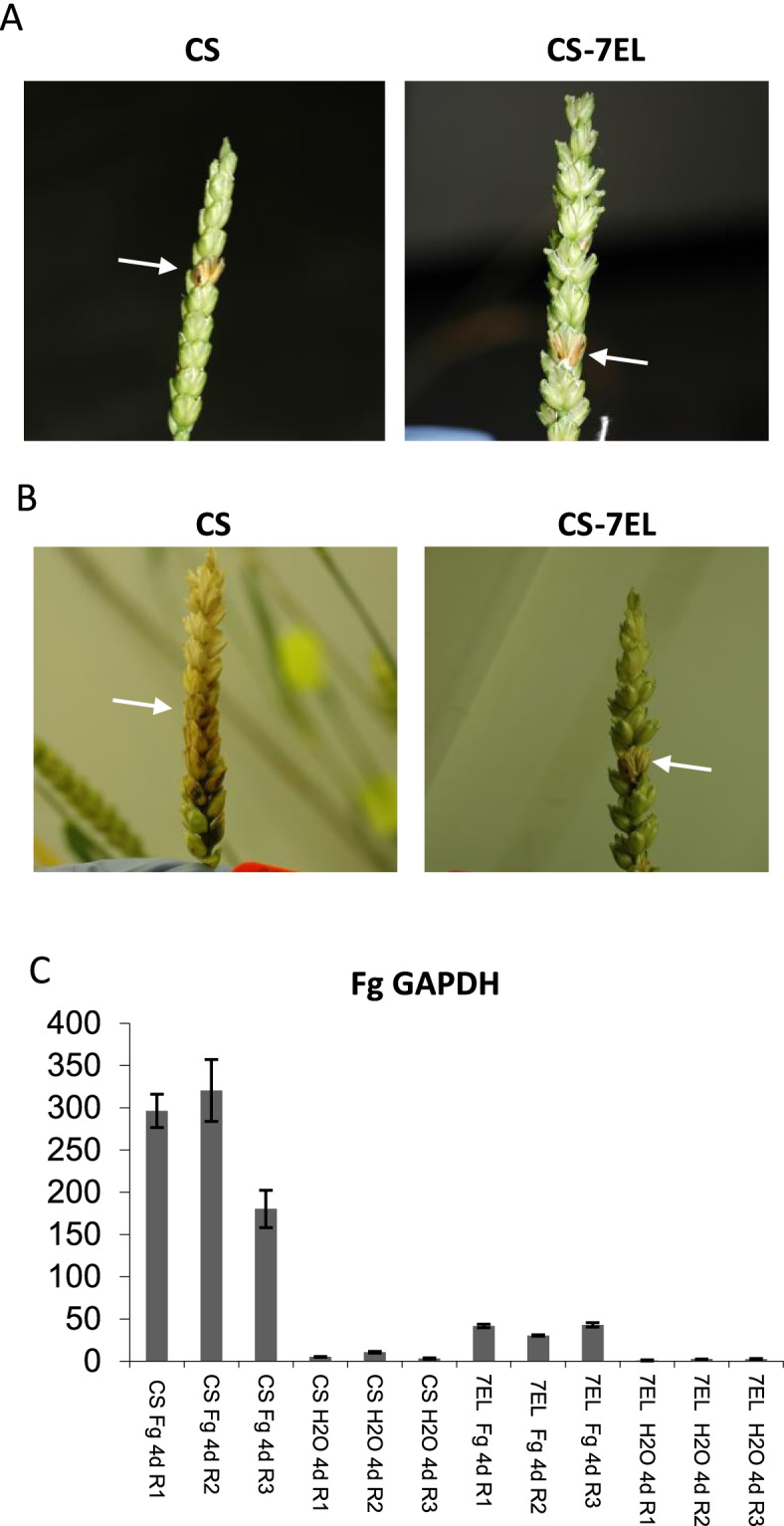
FHB symptoms. A and B: typical examples of CS and CS-7EL spikes at 4 d and 14 d, respectively, after inoculation with *F. graminearum* spores; arrows indicate the inoculated spikelet. C: RT-qPCR analysis showing relative expression (Y-axis) of *F. graminearum* GAPDH transcripts in water- and *F. graminearum*-inoculated rachis samples at 4 d after treatment

**Fig. 4:**
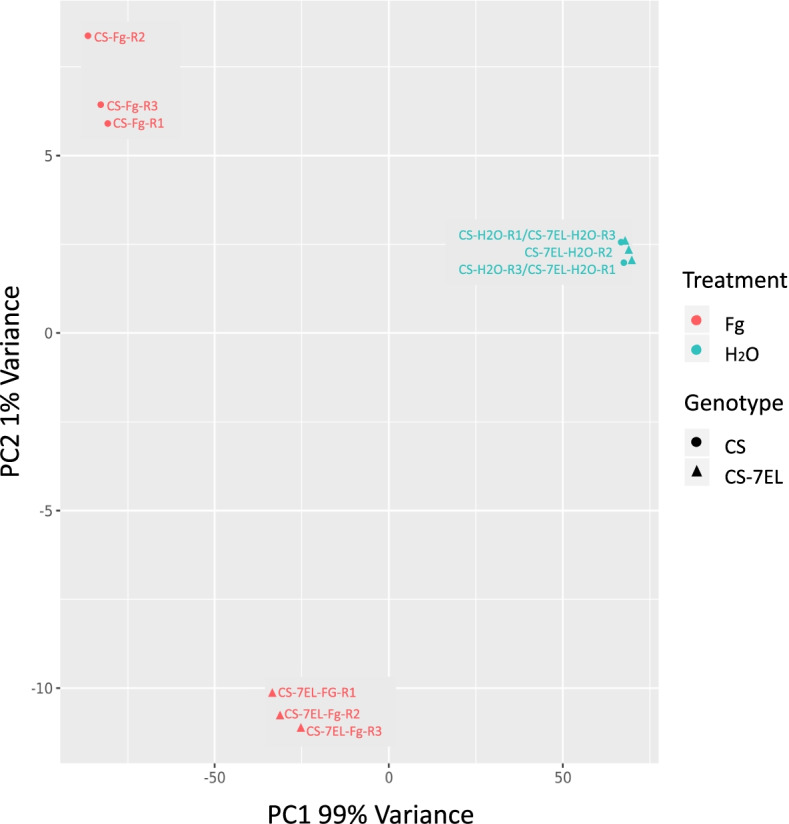
Principal component analysis (PCA) plot. Comparison of global expression profiles in rachis tissues between CS and CS-7EL, 4d after water (mock) and *F. graminearum* (Fg) treatments

In response to *F. graminearum* inoculation, a reduction in browning symptoms can be observed as early as 4 d post inoculation in the infected florets of CS-7EL addition line when compared to CS, and remains visible later in infection (Fig. 3A and B). A previous detailed microscopy study has shown that the largest difference in symptoms was observed in the rachis tissues, with very little spread of the fungus from inoculated florets to adjacent rachis tissues in CS-7EL while abundant spread occurred in CS spikes [9]. This difference became particularly clear and consistent by day 4 after inoculation. Here, we present a comparison of global gene expression profiles between CS and CS-7EL after inoculation with *F. graminearum* or water using rachis tissues sampled at 4 d post inoculation, establishing a reference time point for transcriptomic analyses. Estimation of *F. graminearum* biomass in those rachis samples by quantification of fungal glyceraldehyde-3-phosphate dehydrogenase (GAPDH) transcripts supported the phenotypic observations (Fig. 3C). Principal components analysis of the expression profiles for the wheat genes between CS and CS-7EL revealed that the majority of variation (99%) amongst these samples could be explained by a single principal component (PC1) which correlated with infection (Fig. 4).

**Fig. 5:**
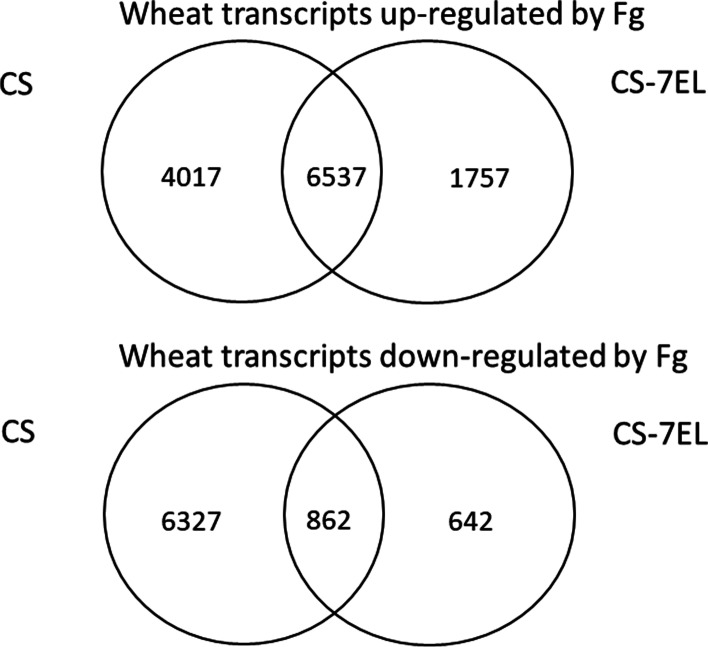
Venn diagram of transcripts. This shows the number of wheat transcripts that were up and down regulated by *F. graminearum* (Fg) infection in rachis tissues of CS and CS-7EL, at 4 d after treatment. Transcripts with log2FC > |2|, padj< 0.001 and normalized counts > 10 for all samples of at least one treatment were considered differentially expressed

A detailed examination of the global expression profiles between water and *F. graminearum* treatments showed that a majority of the wheat transcripts that were significantly (log2FC > |2|, padj< 0.001) up-regulated by the *F. graminearum* treatment were common between CS and CS-7EL (Fig. 5). About 93% (4671/5023) of those common wheat transcripts showed a muted differential expression (log2FC value difference > ǀ1.0ǀ) in CS-7EL when compared to CS, consistent with a tempered response due to a lessened infection (Additional file 11). Validation of expression profiles of select transcripts by RT-qPCR analysis showed similar profile patterns to those observed by RNA-Seq analysis, including transcripts for a WRKY79 (MSTRG.145180), a glucosyltransferase (MSTRG.21806), and a zinc finger protein ZAT (MSTRG.60974) (Additional file 12).

A much smaller number (346/5023) of the common wheat transcripts were up-regulated by *F. graminearum* infection at higher level in CS-7EL than in CS. Among the 146 transcripts with an annotated function in that group, there was a higher proportion of transcripts annotated as pathogenesis-related genes; in particular, glucan endo-1,3-beta-glucosidases, chitinases and peroxidases represented 15, 5 and 3% respectively of those annotated transcripts (Additional file 11). In contrast, among the 1502 annotated common transcripts up-regulated at higher level (log2FC > 1.0) by *F. graminearum* in CS, those annotated as glucan endo-1,3-beta-glucosidases and chitinases represented only 0.5 and 0.1%, respectively, of the transcripts; in comparison, peroxidases still composed 2% of those transcripts.

Comparing the patterns of responses of coding and non-coding wheat transcripts, we found that although a lower proportion of the transcripts classified as non-coding were differentially expressed (log2FC > |2|, padj < 0.001) in response to *F. graminearum* infection (4.2, and 6.7% for non-coding vs 8.4 and 12.3% for coding in CS and CS-7EL, respectively), the differentially expressed (padj < 0.001) non-coding transcripts showed a greater average change in expression than the coding ones (20.8-fold vs 8.6-fold, and 51-fold vs 17-fold for CS genes in CS and CS-7EL backgrounds, respectively) (Additional files 13 and 14, first tab).

There was a much larger number of wheat transcripts downregulated by *F. graminearum* infection in CS than in CS-7EL (Fig. 5), with most of the transcripts being significantly downregulated only in CS. In addition, among the downregulated transcripts common to both lines, about 87% were downregulated at a higher level (log2FC value difference > 1.0) in CS than in CS-7EL (Additional file 11). The E3 ubiquitin-protein ligase MSTRTG.70787 was a typical example of that expression pattern (Additional file 12). The complete lists of wheat transcripts significantly expressed differentially between water and *F. graminearum* treatments for CS and CS-7EL are provided in Additional files 13 and 14, first tab. In addition, a chromosome level representation of the differentially expressed wheat transcripts in CS-7EL in response to *F. graminearum* infection is provided in Additional file 15. One can note a void of differentially expressed genes near the centromeres, consistent with the lower gene density in those areas.

The expression profiles of the transcripts originating from the *Th. elongatum* 7EL chromosome arm were also examined. Of the 1518 transcripts with expression level > 10 normalized counts in all samples of at least one treatment, 236 (16%) were significantly differentially expressed (log2FC > |2|, padj< 0.001) between the water and *F. graminearum* treatments in CS-7EL (Fig. 1, lower panel; Additional file 14, first and second tabs). Of those DE transcripts, 85% were upregulated by *F. graminearum* infection, with lncRNAs and secondary metabolism being the most prevalent enriched functional categories (Additional file 16). The 7EL transcript MSTRG.1335, a putative cytochrome P450 monooxygenase with homology to *A. thaliana* P450 CYP72A15, was both a strongly DE transcript (log2FC of − 6.1) and the highest expressed 7EL transcript in *F. graminearum*-infected CS-7EL samples (Additional file 14, first and second tabs).

**Fig. 6:**
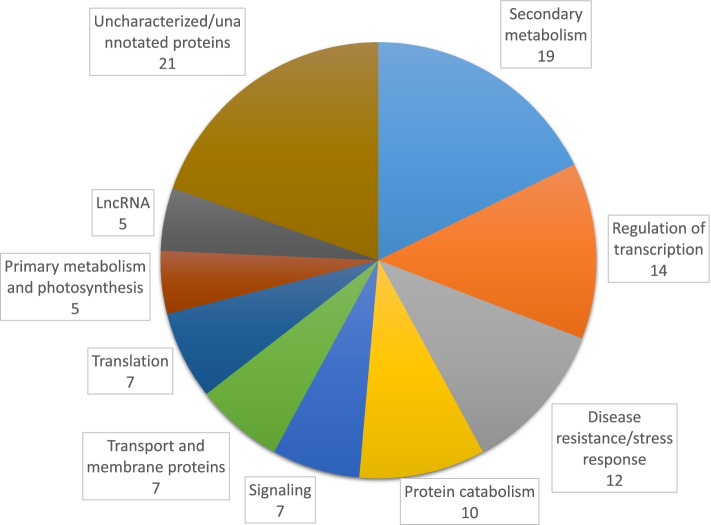
Pie chart representing the number of functionally annotated 7EL transcripts. Transcripts expressed in the rachis and with homology to the 66 Mbp distal end of the *Th. elongatum* 7E from the D-3458 assembly [20] and/or the 27 Mbp distal end of wheat chromosome arm 7DL, were grouped into functional categories

Recent studies have located the FHB resistance locus from *Th. elongatum* chromosome arm 7EL to a fragment located at its distal end [25, 26]. Using 7EL-specific markers, Haldar [26] determined that the 7EL fragment containing the FHB-resistance was homologous to the distal 25 to 27 Mbp of wheat chromosome arm 7DL. Based on the recently published sequence of *Th. elongatum* D-3458 [19], the same 7EL fragment corresponded to the distal 66 Mbp end of the 7E pseudomolecule. The sequence of the 7EL transcripts identified in this study were mapped to Dvorak74 and D-3458 assemblies of *Th. elongatum* as well as to the *T. aestivum* RefSeqv1.0 genomic sequences [17, 20]; a total of 107 7EL transcripts were identified as having their best homology match to either or both the distal 66 Mbp of 7EL from the D-3458 assembly or the distal 27 Mbp of 7DL (Additional file 17). The majority of those transcripts (65%) were expressed at similar levels (log2FC ≤ |2|, padj≥0.001) in the control and *F. graminearum* treatments while 19% were significantly induced by the fungal infection. Of note, about 8% of the transcripts were identified as putative disease resistance proteins, with the majority of them being of the CC-NB-ARC type (Fig. 6, Additional file 17). Alignment of our transcript sequences at the distal end of chromosome arm 7EL with the D-3458 *Th. elongatum* assembly [20] showed homology between 81 and 99%; in addition, a few of our 7EL transcripts had their best match to other chromosomes than 7E in that assembly (Additional file 17).

Finally, we examined the *F. graminearum* transcriptome by comparing infected samples from CS and CS-7EL. Of the 9652 transcripts with expression level > 10 normalized counts for each sample of at least one line, 98% were expressed at a significantly higher level (log2FC > |2|, padj< 0.001) in CS and none were significantly expressed at a higher level in CS-7EL (Additional file 18). These results are consistent with the disease symptoms observed for those two lines. They also indicate that there was no significant change in expression profiles for particular *F. graminearum* genes in response to the FHB resistance in CS-7EL.

## Discussion

Introgression of genes for resistance to rusts and FHB from chromosome arm 7EL of *Th. elongatum* into wheat has been an interest of breeding programs for many years, renewed more recently by more frequent and severe FHB epidemics in many temperate regions of the world. We have sequenced the 7EL chromosome arm from *Th. elongatum* and used the sequence to increase our understanding of the interaction between hexaploid wheat and *Th. elongatum* at the transcriptome level, with a focus on rachis tissues of spikes after mock or *F. graminearum* infection.

The *Th. elongatum* accession used for the production of the CS-7EL telosomic addition line was never specified [15]; however comparisons of the Dvorak74 7EL assembly with the assembly recently published by Wang et al. [19] for accession D-3458 indicate that they are two distinct accessions. The variation catalogue presented here will contribute to expand knowledge of the genetic diversity in *Th. elongatum*.

Our transcriptome analysis of mock control rachis samples has showed that the *Th. elongatum* chromosome arm 7EL contributes to the wheat transcriptome in those CS-7EL tissues, both by expression of 7EL-specific transcripts and by modulating the expression of a subset of wheat transcripts. Further experiments will be required to determine if additional 7EL transcripts are expressed and wheat transcripts modulated in other tissues or treatments of CS-7EL. Our results are consistent with observations by Rey et al. [27], who reported that approximately 3% of the wheat genes in leaves of a CS + barley 7HL addition line were differentially expressed when compared to CS, and that the majority of transcriptional perturbations in an alien introgression are in the introgressed fragment. Dong et al. [28] also observed a significant change in expression levels in 5 and 4%, respectively, of the wheat genes in the CS-*Aegilops longissima* disomic substitution line and disomic addition line that they have analyzed. In both our and their experiments, the DE wheat transcripts were distributed across the whole genome. However, a hot spot of up-regulated wheat transcripts on 7BL was unique to our material. Further experiments will be required to explain this observation.

In the broader context of interspecies hybridization and introgression events between wheat and wild relatives, sequence deletions and epigenetic modifications such as changes in cytosine methylation patterns have been reported; epigenetic changes have been associated with activation of transposons and modifications of wheat gene expression, particularly in the vicinity of the activated transposons [29–32]. Such mechanisms may explain the changes that we have observed in wheat transcript levels.

Many recent studies have shown that lncRNAs play important roles in transcriptional responses to biotic and abiotic stresses (reviewed by [33, 34]). Expression levels of lncRNAs have been shown to be modulated by powdery mildew infection and heat stress [35, 36]. Furthermore, many lncRNAs are co-expressed in pairs with flanking coding genes [37, 38]. Huang et al. [39] showed that a lncRNA with homology to a carboxylesterase-like protein gene is a precursor for a miRNA that is responsible for the nonglaucous phenotype in durum wheat. FHB-responsive lncRNAs were identified in *F. graminearum*-infected barley and proposed to play a role in regulation of the transcriptional response [40]. We found that predicted noncoding RNAs are enriched in the 7EL fragment and overall show more dynamic changes in regulation than predicted coding genes.

Over the last 15 years, many transcriptomic studies have been performed to compare gene expression profiles in FHB-susceptible and –resistant wheat infected by *F. graminearum* (see detailed reviews [41, 42]). A stronger or earlier expression of defense response genes is a recurring pattern observed. Here, in this first, single time-point transcriptome comparison between *F. graminearum*-infected and mock treatment rachis tissues from CS and CS-7EL, respectively susceptible and resistant to FHB, showed wheat DE transcript profiles that were indicative of a more effective defense response to FHB infection in CS-7EL rachis tissues than in those of CS. In particular, many glucan endo-1,3-beta-glucosidases and chitinases were upregulated exclusively in CS-7EL. Glucan endo-1,3-beta-glucosidases (also referred to as beta-1,3-glucanases and PR2) have been shown to release beta-1,3 glucans from plant cell walls, which acts as signal molecules to stimulate the defense response; they also, along with chitinases (PR3), contribute to fungal cell wall degradation [43]. Expression of a beta-1,3-glucanase in FHB-resistant wheat has been previously observed [44, 45]. Also, overexpression of either a beta-1,3-glucanase or a chitinase in transgenic wheat was shown to reduced susceptibility to FHB [46, 47]. In addition, a large proportion of the wheat DE transcripts common between the two lines had a muted response in CS-7EL, suggesting that those transcripts were associated with the level of FHB infection rather than with a successful defense response. Similar types of profiles were observed previously after FHB infection when comparing susceptible and resistant genotypes [48–51].

Differentially expressed transcripts originating from chromosome arm 7EL were enriched in functions associated in general with disease and stress responses, including secondary metabolism, signaling and regulation of transcription. The highly expressed and DE transcript MSTRG.1335 has strong homology to an *Arabidopsis* cytochrome P450 CYP72A15. The function of that cytochrome P450 monooxygenase is not known. Members of the CYP72 group are involved in the metabolism of fairly hydrophobic compounds such as fatty acids and isoprenoids, the catabolism of hormones (brassinosteroids and gibberellin) and the biosynthesis of cytokinins [52]. A wheat cytochrome P450 CYP72A has been shown to enhance resistance to *F. graminearum* mycotoxin deoxynivalenol and contribute to host resistance in wheat [53]. However, MSTRG.1335 is not expected to contribute directly to FHB resistance as it is located outside of the 7EL distal end, starting at 7E position 590,773,845 in D-3458 assembly [20]. Future transcriptomic analyses using earlier and later time points will be needed to complement this study and support its findings.

There was a large difference in size between the distal end of chromosome arm 7EL that carries the gene for FHB resistance and the orthologous region on 7DL. In addition, chromosomal inversions were noted between 7EL and 7DL in that region. Similar observations were made by Wang et al. [19]. They showed that the distal region of 7EL had undergone expansion, particularly for disease resistance gene analogs. Many of the transcripts that we detected in that distal region, irrespective of their response to *F. graminearum* infection, have the potential to contribute to FHB resistance; functions such as NB-ARC domain-containing disease resistance gene analog, receptor protein and LRR receptor-like serine/threonine-protein kinases, cytochrome P450 monooxygenase, and callose synthase have been implicated in disease resistance in other pathosystems [54–57].

A gene for FHB resistance was previously mapped to the distal end of chromosome arm 7EL in the closely related species *Th. ponticum* [58]. Wang et al. [19] have proposed a candidate gene for that resistance, a unique glutathione S-transferase (GST) that appears to have originated by horizontal transfer from a fungal species. A similar GST transcript, MSTRG.130, was present in the distal end of our 7EL chromosome arm, and it was strongly upregulated by *F. graminearum* infection. MSTRG.130 is located on a scaffold that was not anchored during reference-guided assembly. Notably, MSTRG.130 has marked differences from the proposed FHB7 GST sequence with a large insertion present in MSTRG.130 that is absent in the gene. Manual inspection of Dvorak74 derived reads mapped to the FHB7 gene in the D-3458 assembly showed several differences, including the presence of a subpopulation of reads carrying an insertion and two or more distinct populations of reads. As the CS-7EL addition line was derived from a single 7EL telosome, the presence of two or more transcripts is consistent with more than one copy of the gene relative to the GST identified by Wang et al. [19]. Further experiments will be required to determine if the product of MSTRG. 130 and/or one of the other transcripts in the distal end of 7EL contribute to FHB resistance in *Th. elongatum*.

## Conclusions

Genomic sequencing of the chromosome arm 7EL from an unidentified *Th. elongatum* accession provided a first assessment of genetic diversity in that species for a chromosomal region that contains sought after resistance to important wheat diseases, including FHB, leaf rust and stem rust. Impact of 7EL on the wheat transcriptome and contribution of 7EL to that transcriptome were demonstrated in a comparison between rachis tissues treated with water or *F. graminearum*. A list of expressed genes from the distal region of 7EL containing a locus for FHB resistance was established. This novel information will contribute to identify the gene(s) contributing to the strong FHB resistance in that region and develop strategies to facilitate its transfer to wheat.

## Material and methods

### Plant material

Genotypes used in this work included the FHB-susceptible cultivar Chinese Spring (CS) and the CS-*Th. elongatum* ditelosomic addition line CS-7EL containing the long arm of the 7E chromosome from a diploid accession of *Th. elongatum* [15]. Seeds from CS-7EL were kindly supplied by Dr. Mingcheng Luo (University of California, Davis, USA) and have been deposited at Plant Gene Resources of Canada (Saskatoon, Canada). CS-7EL genetic content was revisited by [59]. Presence of 7EL DNA in the addition line was confirmed in each experiment using 7E-specific markers [7] (data not shown).

Pictures of infected CS and CS-7EL heads were taken at 4d and 14d after inoculation using a camera Nikon D70. GISH analysis was performed as described in [60], using total genomic DNA from *Th. elongatum* labelled with fluorescein-12-dUTP for the hybridization step.

### Flow-sorting of *Th. elongatum *7EL and paired-end library construction

Seeds of CS-7EL were germinated, their meristem root-tip cells were synchronized and used to prepare suspensions of intact mitotic metaphase chromosomes [13]; GAA microsatellites on chromosomes were labelled by FITC [61] and chromosomal DNA was stained by DAPI. The samples were sorted in four independent batches using a FACSAria II SORP flow cytometer and sorter (Becton Dickinson Immunocytometry Systems, San José, USA); bivariate analysis GAA-FITC vs. DAPI was used to discriminate the population representing 7EL (Additional file 19). Chromosomal DNA was amplified individually using an Illustra GenomiPhi DNA amplification kit (GE Healthcare, Mississauga, Canada) following [62]. Four Truseq PCR-free (Illumina, San Diego, CA) paired-end libraries with varying insert sizes were prepared from a single pool of amplified DNA.

### Mate pair library construction

For mate pair library construction, nuclear DNA from CS + 7EL seedlings was purified as described in [63] with the addition of a centrifugation step (2 min, 55 rcf) to remove cellular debris after the first resuspension of nuclei. All mate pair libraries aside from the 40 kb library were prepared with the Nextera MP kit (Illumina, San Diego, CA) using the gel-based protocol with the following modifications. Two pairs of tagmentation reactions with 16 μL of tagment enzyme and 6 or 8 μg of nuclear DNA were incubated in 400 μL reactions. Pairs of reactions were pooled and separated by field inversion gel electrophoresis (0.6% Megabase gel, 14.5 h). The size-resolved DNA was divided into 12 fractions and purified using a Zymoclean large fragment DNA recover kit (Zymo Research, Irvine, CA) according to manufacturer’s instructions. Five of the fractions gave a yield greater than 600 ng and were divided for subsequent steps to make distinct libraries. Libraries were amplified with 10, 12 or 15 cycles on the basis of the insert size and amount of DNA used for circularization. The 40 kb mate pair library was constructed using the NxSeq 40 kb Mate Pair Cloning Kit (Lucigen, Middleton, WI) according to manufacturer’s instructions.

### Genomic sequencing, read processing and assembly

All sequencing was performed at the National Research Council of Canada’s Nucleic Acid Solutions facility on an Illumina Hiseq 2500. Truseq paired end, Truseq PCRfree and Nextera mate pair reads were trimmed for quality and adapter sequences with Trimmomatic v0.30 [64] using options PE –phred33 Crop:150 Leading:20 Trailing:20 Slidingwindow:15 Minlen:50 Illuminaclip:1:40:15. PhiX spike-in was removed with Bowtie2 [65] with options --no-discordant --no-mixed -k1 --very-fast --un-conc. NxSeq libraries trimming also included a Headcrop:15 option for Trimmomatic. Replicate lanes of data were combined for each library and duplicate reads were removed using a custom Perl script and FastUniq [66] with default options. Complete junction sequences for Nextera and NxSeq libraries were identified using a custom perl script that removed the junction and any post junction sequence within a read. Residual incomplete Nextera junctions were removed from the 3′-ends of reads with Trimmomatic v0.30 using options PE -phred33 Illuminaclip:NexteraJunction.fasta:1:3:3 Minlen:32. Only the reads with a confirmed junction sequence, BfaI restriction site CTAG, were used from the NxSeq library.

The initial assembly of 7EL was generated using all paired end reads using Ray [14] with kmer length of 69. This initial assembly was supplemented with 7EL reads from mate libraries generated using genomic DNA from the CS-7EL addition line. As these libraries contained a mixture of 7EL and wheat sequences, individual mate pair reads were mapped against each chromosome (arm) of the IWGSC Survey sequence v 1.0 with Bowtie v1.0.0 [67] using the option -m 1. Reads were retained if both pairs mapped exclusively within a single chromosome arm.

Mate-pair libraries are often contaminated with paired-end sequences. Based on comparison of mapping of the CS mates to the chromosome 3B reference [68] in forward-reverse vs reverse-forward orientations (Additional files 20 and 21), we concluded that there was very limited paired-end contamination in all but two of the Nextera libraries. These two libraries also showed low diversity and were excluded from further analysis. We used all mate pair sequences for the remaining libraries regardless of whether a junction was identified during read processing. We did not use mate pairs that did not have a junction sequence identified from the 40 kb fosmid-based libraries.

Scaffolding of the processed mate library reads was performed with SSPACE Standard v3.0 (BaseClear, The Netherlands) using the following options: -b5 -bowtie. Gapfilling was then completed with SOAPdenovo2’s GapCloser [69] using the paired-end libraries and applying default parameters; for gapfilling of wheat scaffolds, we used the paired end data associated with the IWGSC RefSeq v1.0 [17]. BUSCO v3 [16] in mode “genome” was used to assess the completeness of the 7EL genome assembly and annotation.

### Reference-guided Dvorak74 7EL assembly and comparison with D-3458 assembly

The D-3458 assembly, ASM1179987v1 [20], was obtained from NCBI and used to guide assembly of the Dvorak74 7EL draft scaffolds using Ragtag v1.1.1 [21] and parameters -f 500 and -q5. The reference guided Dvorak74 7EL assembly was compared to the D-3458 assembly using Nucmer v3.1 [70] with parameters -l200 and -c500, structural variants were identified using Assemblytics [71] and converted to vcf format using the convertAssemblytics function of Survior (v1.0.7) [72]. Annotations from the D-3458 assembly were lifted over to the final Dvorak74 7EL assembly using liftoff v1.5.1 [73] with default parameters and compared with de novo annotations using bedtools (v2.29.2) intersect with the -ba option [74].

### Gene profiling experiment and RNA-seq data analysis

Plant growth conditions, fungal culture and wheat head inoculation with *F. graminearum* strain DAOM 180378 (Canadian Collection of Fungal Cultures, Agriculture and Agri-Food Canada, Ottawa, Canada) were described in detail in [75]. CS and CS-7EL rachis from *F. graminearum*-inoculated or water-treated wheat heads were harvested at 4d after treatment; three replicates each including ten to twelve heads were completed per treatment. Plant total RNA isolation from rachis tissues and multiplex cDNA libraries for RNA-seq were prepared as described in [75]. The library for the water-treated CS replicate 2 was found to be misidentified and was not used for the analysis in this paper.

For mapping of RNA-seq reads, a single combined reference consisting of the genomic sequences of wheat (RefSeq V1.0 [17]), 7EL (developed as part of this study) and *F. graminearum strain* DAOM180378 [76] was used with STAR [77] v2.4.2a using the options outFilterMultimapNmax 10, outFilterMismatchNoverLmax 0.02, alignIntronMin 10, alignIntronMax 12,000, outFilterMultimapScoreRange 0, outFilterMatchNminOverLread 0.9. Transcripts models were generated and merged using Stringtie [78]. Per transcript read counts were calculated with HTseq [79] v0.6.1 using the options —stranded = reverse and —-order = pos.

Differential expression analysis was done using DESeq2 [80]; DE transcripts with log2FC > |2|, padj< 0.001 (using the Benjamini -Hochberg multiple testing correction [81]) and normalized counts > 10 for all samples of at least one line or treatment are presented in Additional files 8, 11, 13, 14, 18. 7EL transcripts with normalized counts > 10 for all samples of at least one treatment are presented in Additional file 14.

To obtain functional annotation, transcript models were mapped using BLASTX against UniProtKB/Swiss-Prot and Reference proteins databases at NCBI [82], and Araport11 protein sequences at The Arabidopsis Information Resource [83]. GO enrichment was performed using TopGO [84].

Long non-coding RNAs (lncRNAs) were identified across CS, 7EL and *F. graminearum* transcripts with CNIT ([23], version 2019-1-1), using the plant model (−m pl), and with CPC2 using default parameters ([24], version 2017-03-6).

### RT-qPCR assays

Total RNA was cleaned up, cDNA synthesized, fungal biomass estimation and RT-qPCR assays for wheat genes were performed as described in Pan et al. [50]. Wheat gene selection for validation was done using similar criteria as in [50]. Gene-specific RT-qPCR primers were designed using the free online OligoAnalyzer (Integrated DNA Technologies [85]). All primers are listed in Additional file 22. The results were analysed as described in Pan et al. [50], except that GAPDH (TraesCS7A01G313100) was used instead of AOx (TraesCS2A01G327600) as the third reference wheat gene for normalisation of the data.

## Supplementary Information

**Additional file 1.**

**Additional file 2.**

**Additional file 3.**

**Additional file 4.**

**Additional file 5.**

**Additional file 6.**

**Additional file 7.**

**Additional file 8.**

**Additional file 9.**

**Additional file 10.**

**Additional file 11.**

**Additional file 12.**

**Additional file 13.**

**Additional file 14.**

**Additional file 15.**

**Additional file 16.**

**Additional file 17.**

**Additional file 18.**

**Additional file 19.**

**Additional file 20.**

**Additional file 21.**

**Additional file 22.**

## Data Availability

The raw reads and assembled 7EL genomic sequence are available from NCBI BioProject PRJNA450404 [86]. RNA-seq data generated in this study is available in the NCBI Gene Expression Omnibus under accession GSE70797 [87]. Analyzed data are available as Additional files to this article.

## Abbreviations

CS: Chinese Spring wheat cultivar
CS-7EL: CS addition line containing a large fragment from the long arm of the chromosome 7E of *Thinopyrum elongatum*
DE: Differentially expressed
FC: Fold change
FHB: Fusarium head blight
lncRNA: Long non-coding RNA
NCBI: National Center for Biotechnology Information
