# Supplementary material for: Genomic sequencing of Thinopyrum elongatum chromosome arm 7EL, carrying fusarium head blight resistance, and characterization of its impact on the transcriptome of the introgressed line CS-7EL
Source: BMC Genomics. 2022 Mar 23;23:228. doi: 10.1186/s12864-022-08433-8 (PMC8944066; doi:10.1186/s12864-022-08433-8)
Supplement: Supplementary file 1 — Additional file 1. [file 12864_2022_8433_MOESM1_ESM.pptx]

## Slide 1
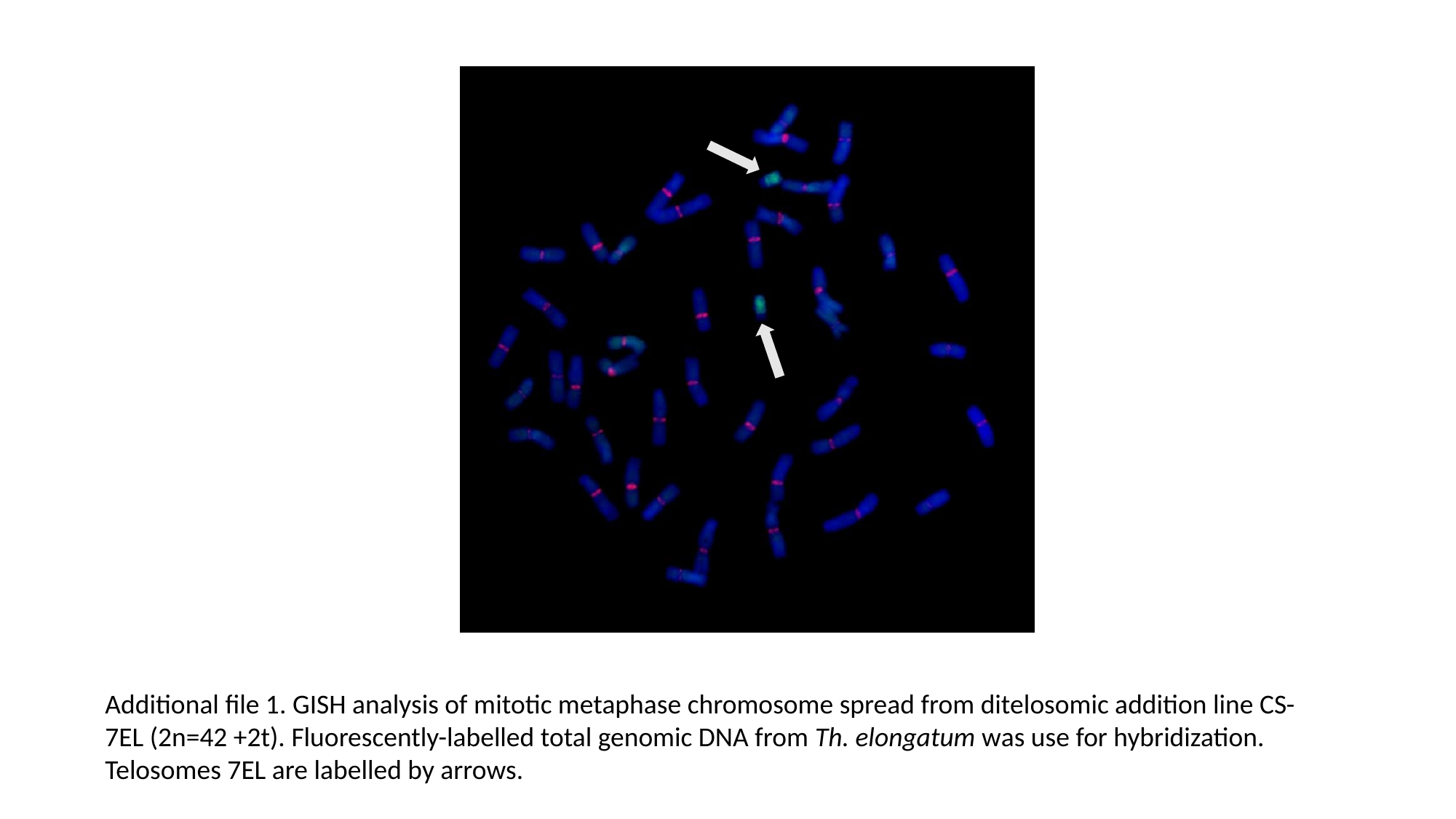

Additional file 1. GISH analysis of mitotic metaphase chromosome spread from ditelosomic addition line CS-7EL (2n=42 +2t). Fluorescently-labelled total genomic DNA from Th. elongatum was use for hybridization. Telosomes 7EL are labelled by arrows.
