# Supplementary material for: Genomic sequencing of Thinopyrum elongatum chromosome arm 7EL, carrying fusarium head blight resistance, and characterization of its impact on the transcriptome of the introgressed line CS-7EL
Source: BMC Genomics. 2022 Mar 23;23:228. doi: 10.1186/s12864-022-08433-8 (PMC8944066; doi:10.1186/s12864-022-08433-8)
Supplement: Supplementary file 3 — Additional file 3. [file 12864_2022_8433_MOESM3_ESM.docx]

Additional file 3. Assessment of the completeness, expressed as number of orthologs, of the genome assembly and annotation for the 7EL chromosome fragment, and for matched segments from chromosomes 7A, 7B and 7D of the wheat refseq v1.0 assembly [17], using BUSCO v3 [16]. A total of 3278 near-universal single-copy orthologs were used for the assessments.

| BUSCO category | 7EL | 7A | 7B | 7D |
| --- | --- | --- | --- | --- |
| Complete and single copy | 211 | 242 | 190 | 271 |
| Complete and duplicated | 20 | 2 | 6 | 2 |
| Fragmented | 23 | 16 | 16 | 16 |
| Missing | 3024 | 3018 | 3066 | 2989 |
