# Supplementary figures and images for: Genomic sequencing of Thinopyrum elongatum chromosome arm 7EL, carrying fusarium head blight resistance, and characterization of its impact on the transcriptome of the introgressed line CS-7EL
Source: BMC Genomics. 2022 Mar 23;23:228. doi: 10.1186/s12864-022-08433-8 (PMC8944066; doi:10.1186/s12864-022-08433-8)

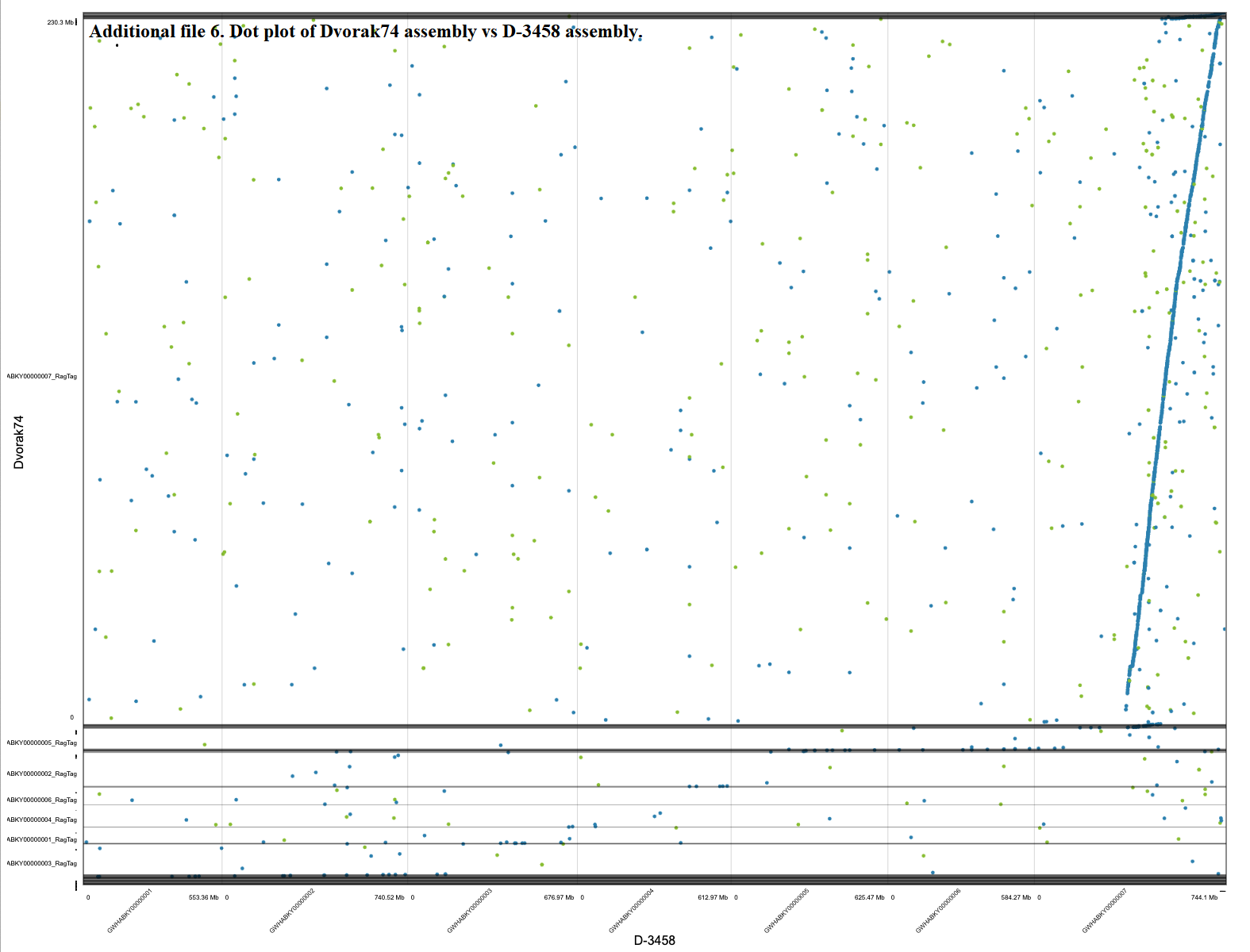

Supplement: Supplementary file 6 — Additional file 6. [file 12864_2022_8433_MOESM6_ESM.tif]

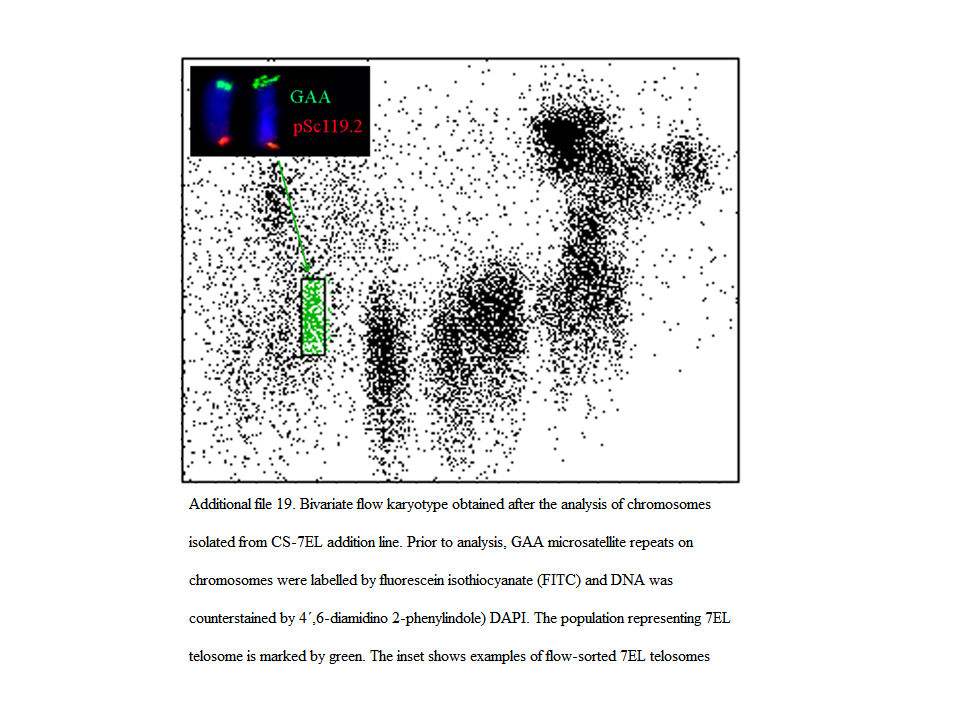

Supplement: Supplementary file 19 — Additional file 19. [file 12864_2022_8433_MOESM19_ESM.tif]
