# Supplementary material for: Genomic sequencing of Thinopyrum elongatum chromosome arm 7EL, carrying fusarium head blight resistance, and characterization of its impact on the transcriptome of the introgressed line CS-7EL
Source: BMC Genomics. 2022 Mar 23;23:228. doi: 10.1186/s12864-022-08433-8 (PMC8944066; doi:10.1186/s12864-022-08433-8)
Supplement: Supplementary file 9 — Additional file 9. [file 12864_2022_8433_MOESM9_ESM.pptx]

## Slide 1
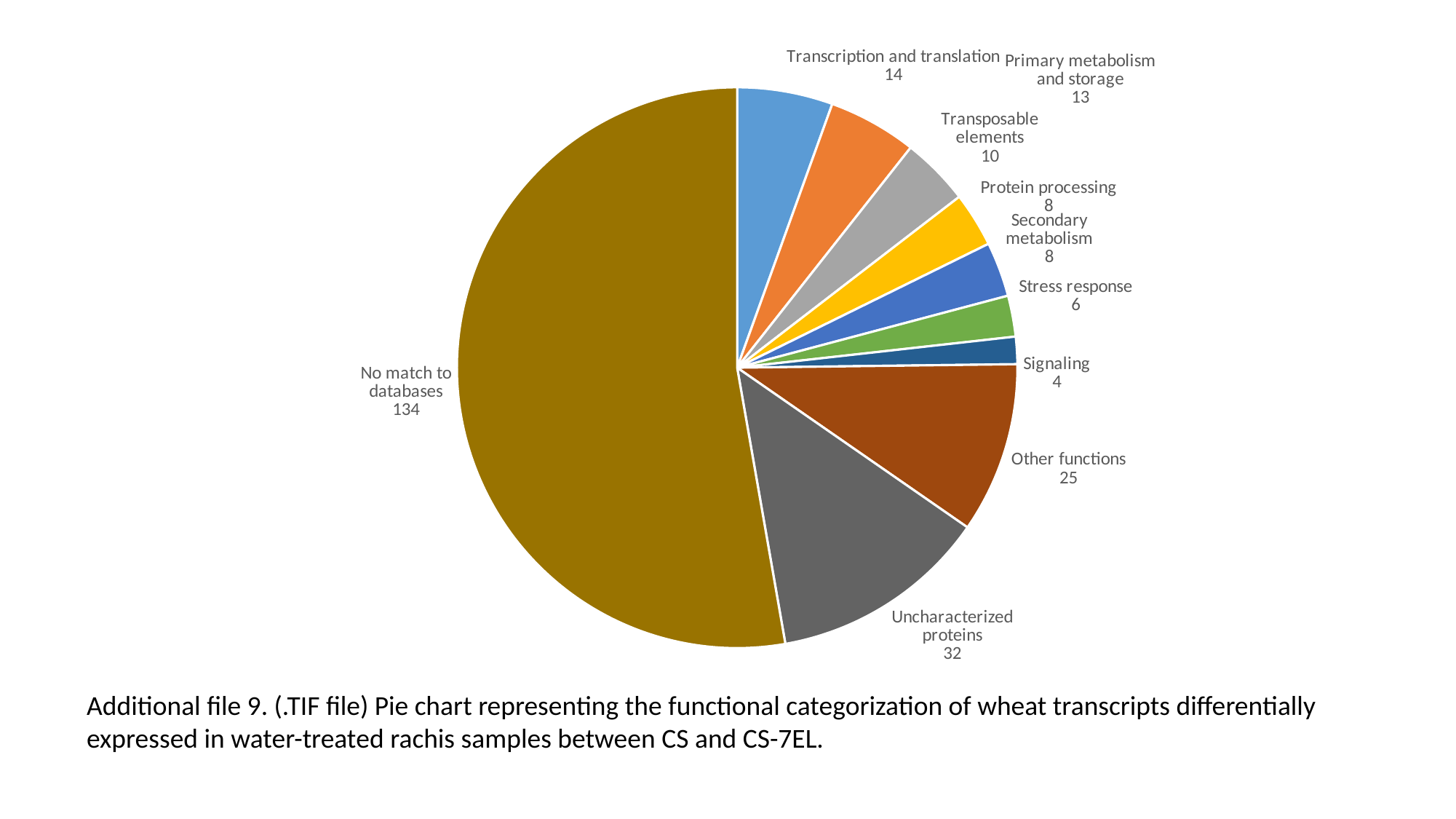

### Chart
| Category | 28 |
|---|---|
| Transcription and translation | 14.0 |
| Primary metabolism and storage | 13.0 |
| Transposable elements | 10.0 |
| Protein processing | 8.0 |
| Secondary metabolism | 8.0 |
| Stress response | 6.0 |
| Signaling | 4.0 |
| Other functions | 25.0 |
| Uncharacterized proteins | 32.0 |
| No match to databases | 134.0 |Additional file 9. (.TIF file) Pie chart representing the functional categorization of wheat transcripts differentially expressed in water-treated rachis samples between CS and CS-7EL.
