# Supplementary material for: Genomic sequencing of Thinopyrum elongatum chromosome arm 7EL, carrying fusarium head blight resistance, and characterization of its impact on the transcriptome of the introgressed line CS-7EL
Source: BMC Genomics. 2022 Mar 23;23:228. doi: 10.1186/s12864-022-08433-8 (PMC8944066; doi:10.1186/s12864-022-08433-8)
Supplement: Supplementary file 10 — Additional file 10. [file 12864_2022_8433_MOESM10_ESM.pptx]

## Slide 1
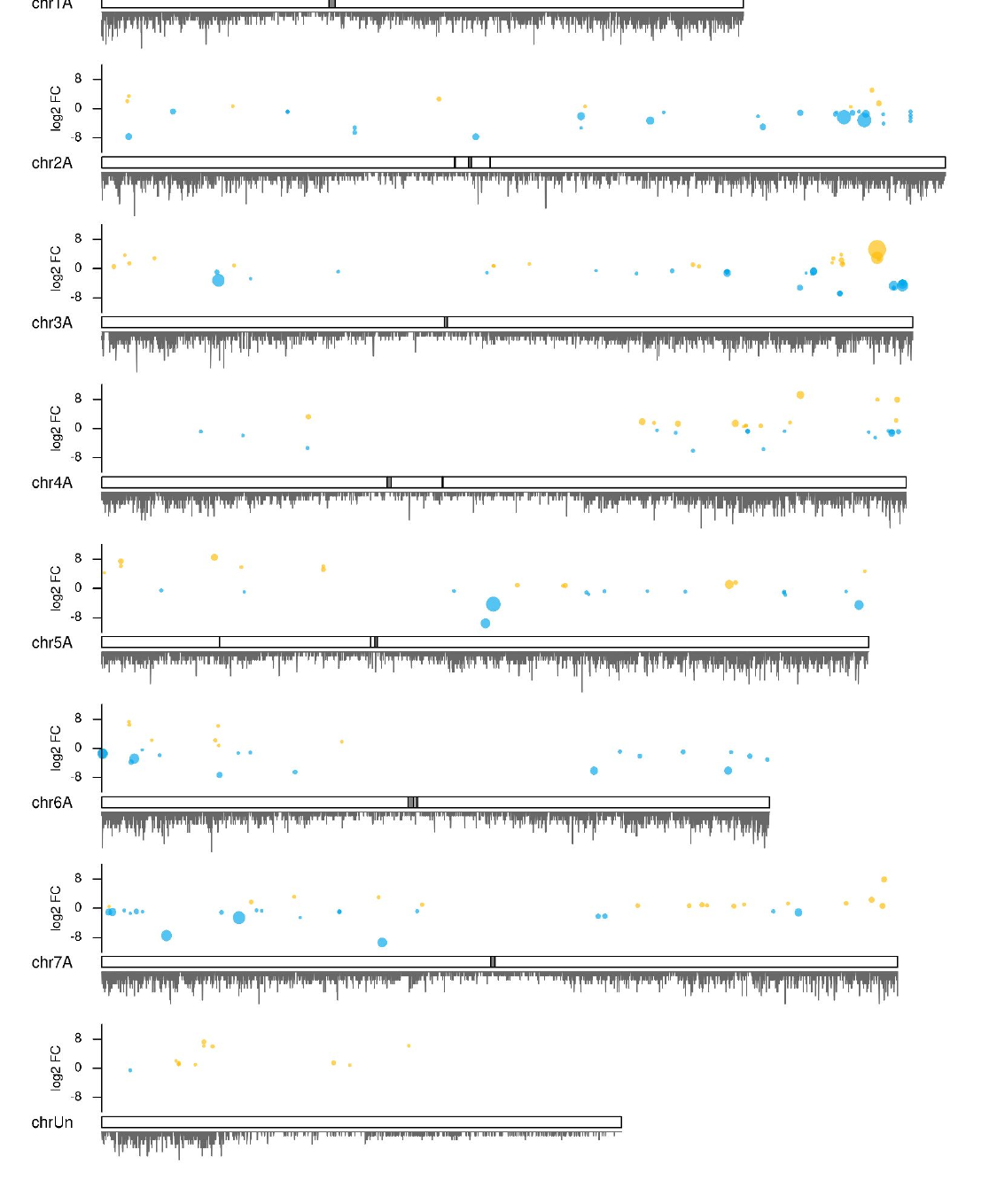

## Slide 2
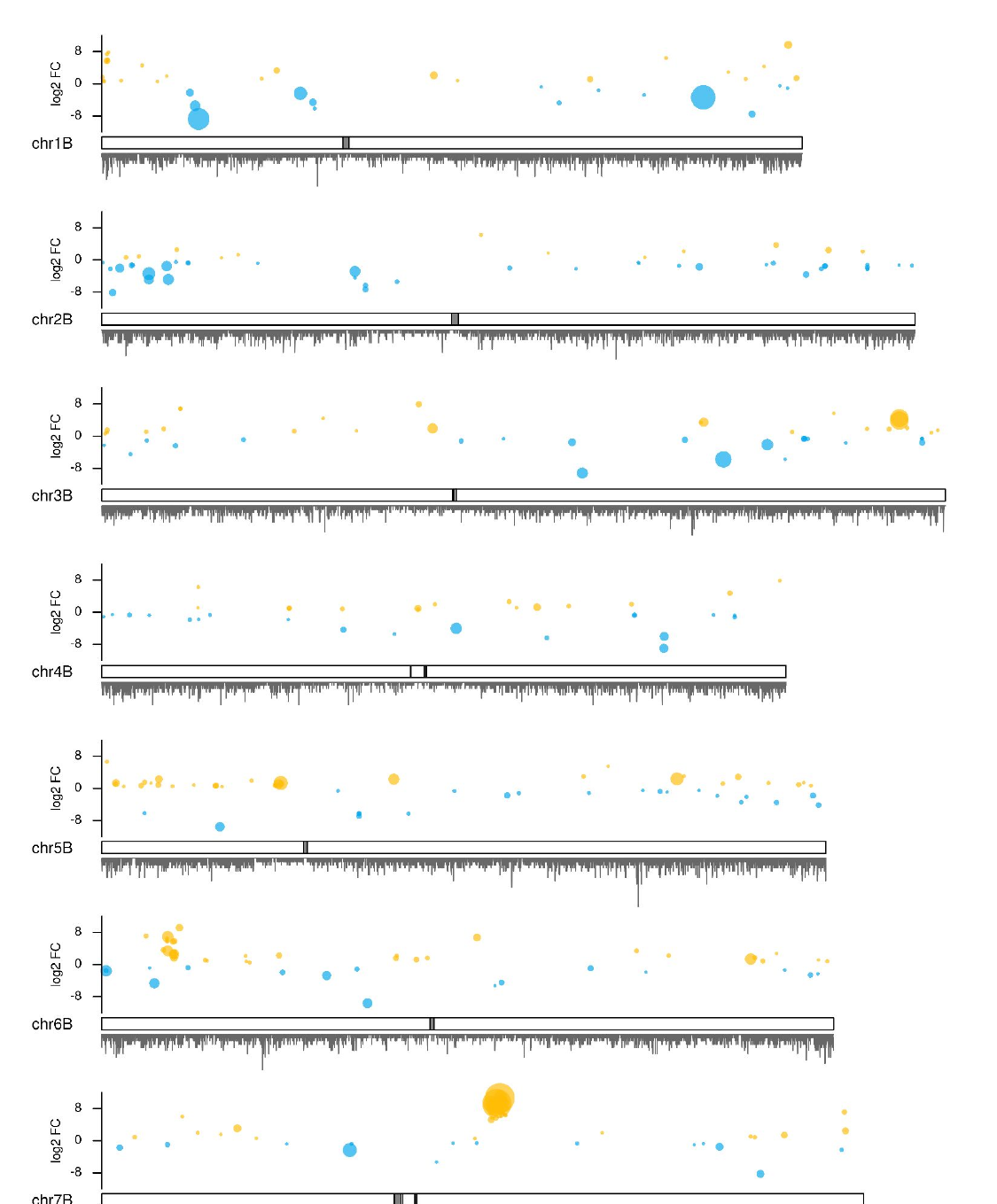

## Slide 3
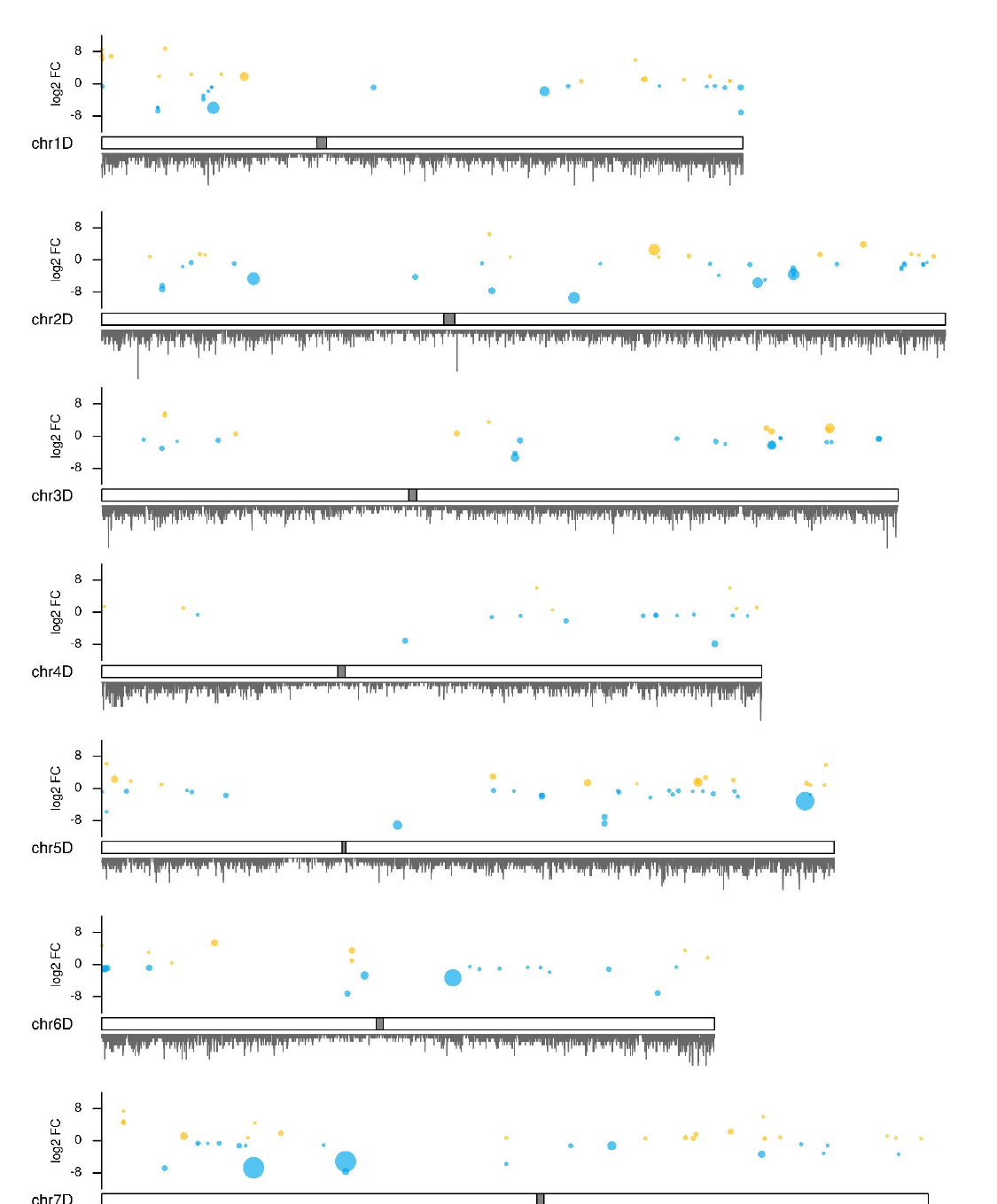

## Slide 4
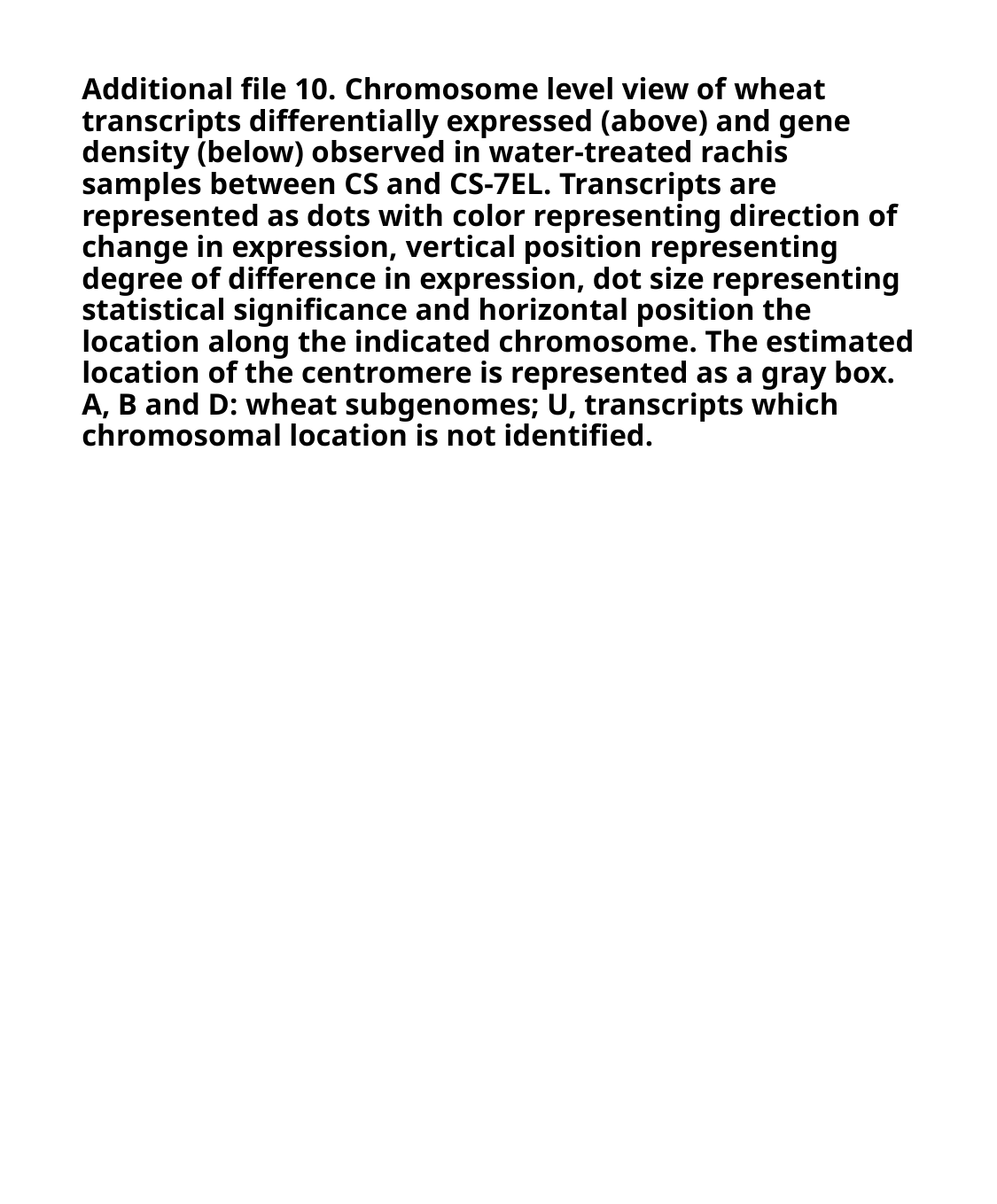

# Additional file 10. Chromosome level view of wheat transcripts differentially expressed (above) and gene density (below) observed in water-treated rachis samples between CS and CS-7EL. Transcripts are represented as dots with color representing direction of change in expression, vertical position representing degree of difference in expression, dot size representing statistical significance and horizontal position the location along the indicated chromosome. The estimated location of the centromere is represented as a gray box. A, B and D: wheat subgenomes; U, transcripts which chromosomal location is not identified.
