# Supplementary material for: Genomic sequencing of Thinopyrum elongatum chromosome arm 7EL, carrying fusarium head blight resistance, and characterization of its impact on the transcriptome of the introgressed line CS-7EL
Source: BMC Genomics. 2022 Mar 23;23:228. doi: 10.1186/s12864-022-08433-8 (PMC8944066; doi:10.1186/s12864-022-08433-8)
Supplement: Supplementary file 12 — Additional file 12. [file 12864_2022_8433_MOESM12_ESM.pptx]

## Slide 1
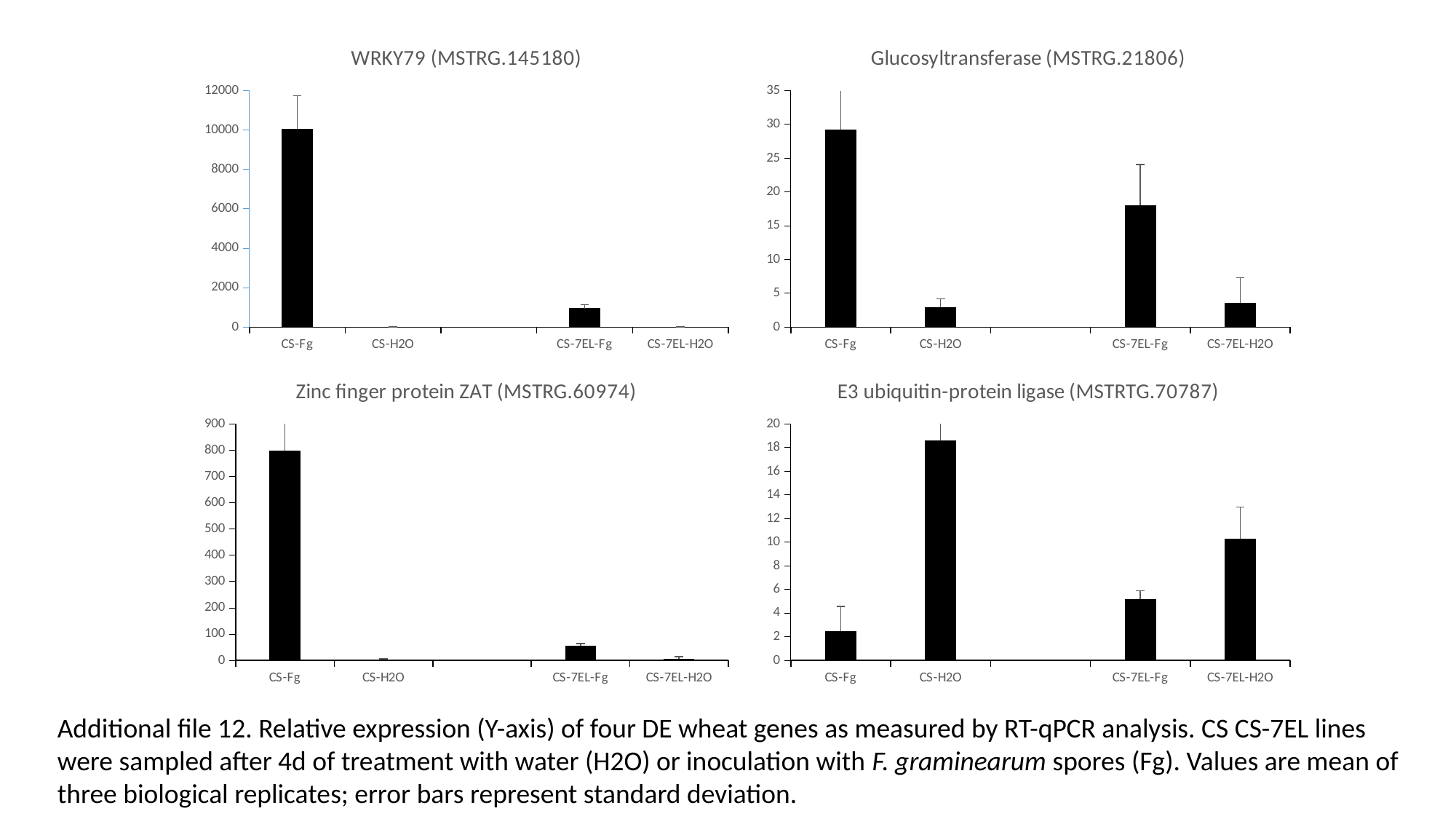

### Chart: WRKY79 (MSTRG.145180)
| Category | |
|---|---|
| CS-Fg | 10043.6 |
| CS-H2O | 2.03 |
| | None |
| CS-7EL-Fg | 945.2 |
| CS-7EL-H2O | 1.8 |
### Chart: Glucosyltransferase (MSTRG.21806)
| Category | |
|---|---|
| CS-Fg | 29.295 |
| CS-H2O | 2.985 |
| | None |
| CS-7EL-Fg | 18.015 |
| CS-7EL-H2O | 3.615 |
### Chart: Zinc finger protein ZAT (MSTRG.60974)
| Category | |
|---|---|
| CS-Fg | 797.6421743795432 |
| CS-H2O | 4.684893830193097 |
| | None |
| CS-7EL-Fg | 57.05559792110281 |
| CS-7EL-H2O | 7.225247059471279 |
### Chart: E3 ubiquitin-protein ligase (MSTRTG.70787)
| Category | |
|---|---|
| CS-Fg | 2.495669924437002 |
| CS-H2O | 18.590036014272023 |
| | None |
| CS-7EL-Fg | 5.203345763714014 |
| CS-7EL-H2O | 10.288773454025499 |Additional file 12. Relative expression (Y-axis) of four DE wheat genes as measured by RT-qPCR analysis. CS CS-7EL lines were sampled after 4d of treatment with water (H2O) or inoculation with F. graminearum spores (Fg). Values are mean of three biological replicates; error bars represent standard deviation.
