# Supplementary material for: Genomic sequencing of Thinopyrum elongatum chromosome arm 7EL, carrying fusarium head blight resistance, and characterization of its impact on the transcriptome of the introgressed line CS-7EL
Source: BMC Genomics. 2022 Mar 23;23:228. doi: 10.1186/s12864-022-08433-8 (PMC8944066; doi:10.1186/s12864-022-08433-8)
Supplement: Supplementary file 15 — Additional file 15. [file 12864_2022_8433_MOESM15_ESM.pdf]

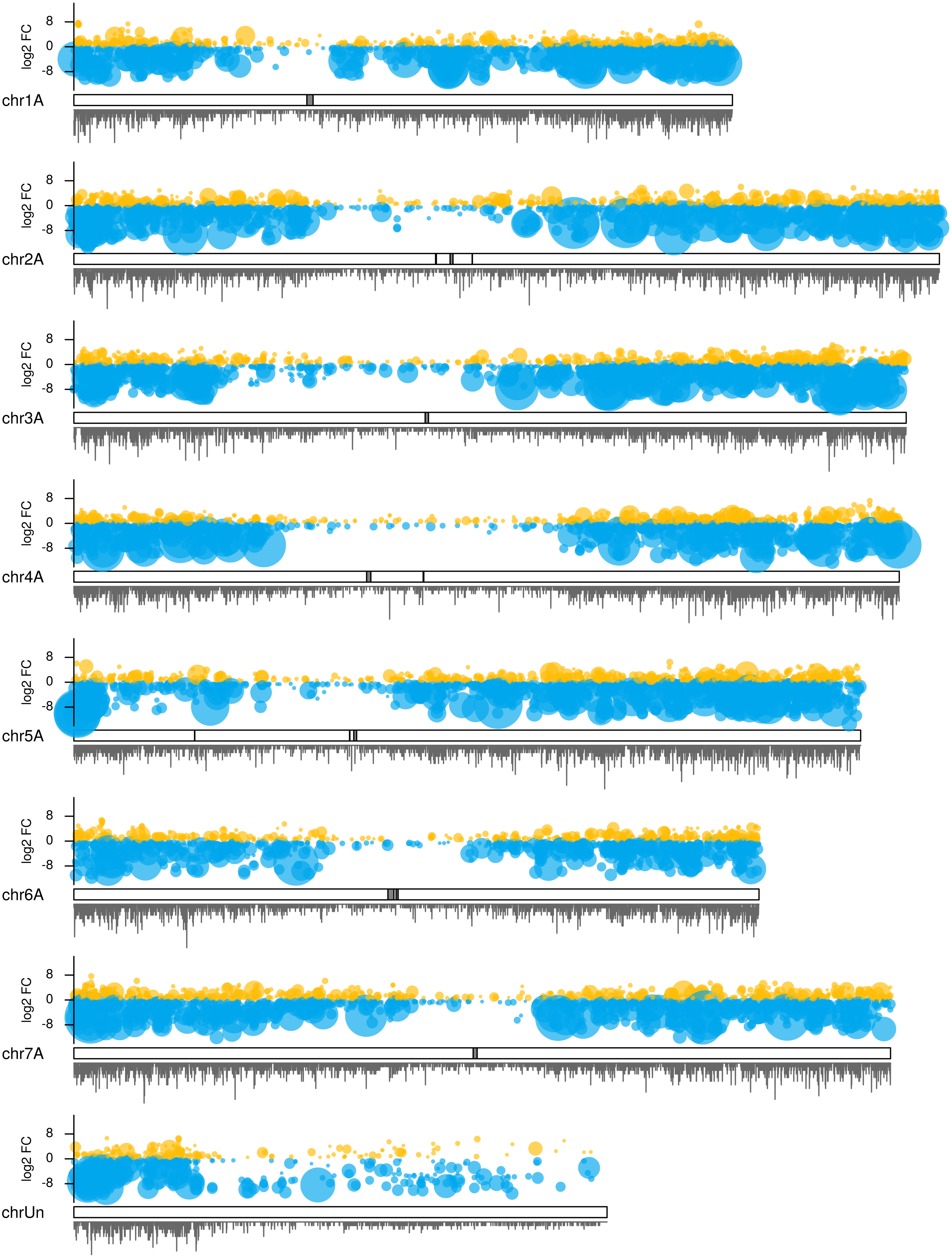
log2 FC
8
0
-8
chr1A
chr2A
chr3A
chr4A
chr5A
chr6A
chr7A
chrUn

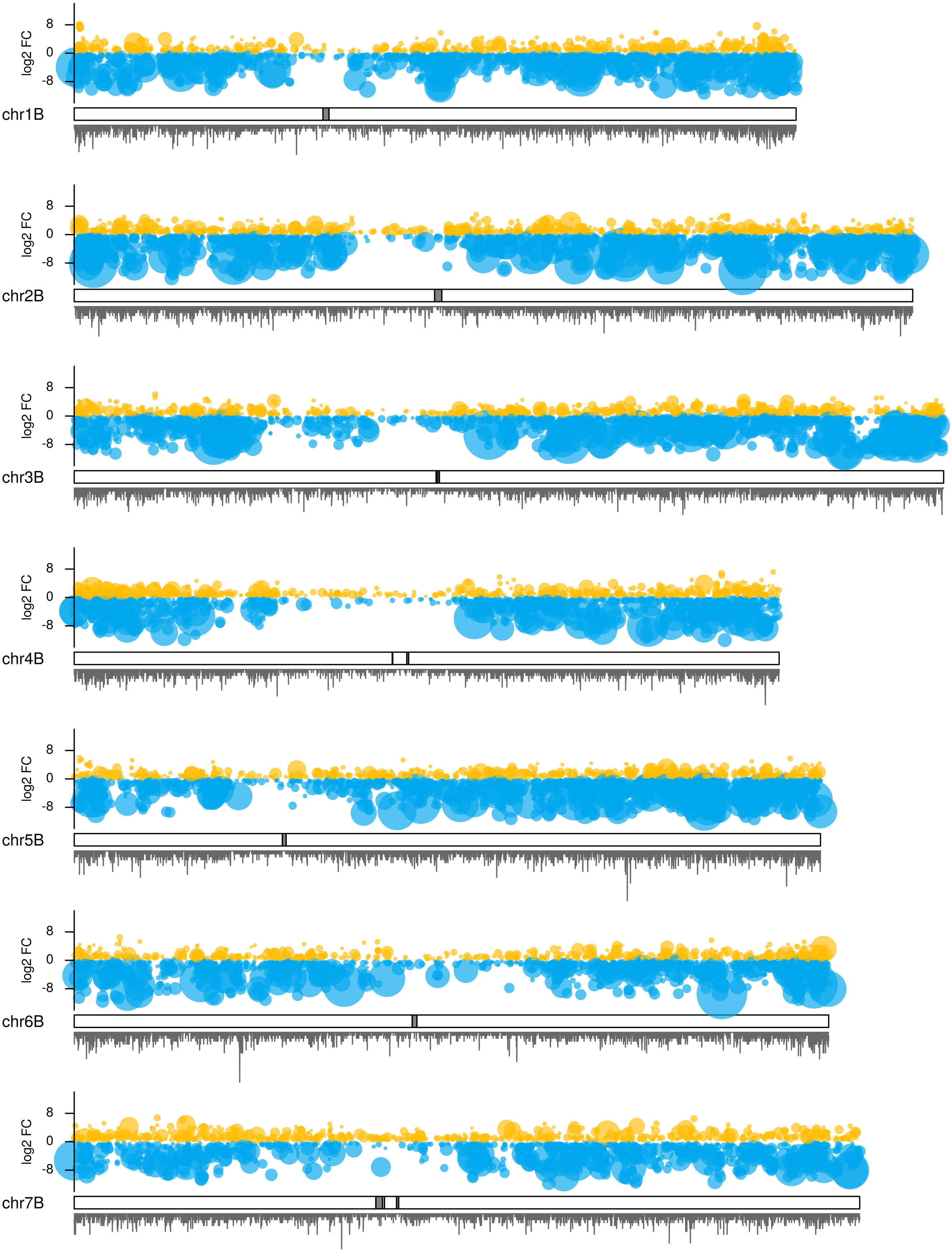
log2 FC
8
0
-8
chr1B
chr2B
chr3B
chr4B
chr5B
chr6B
chr7B

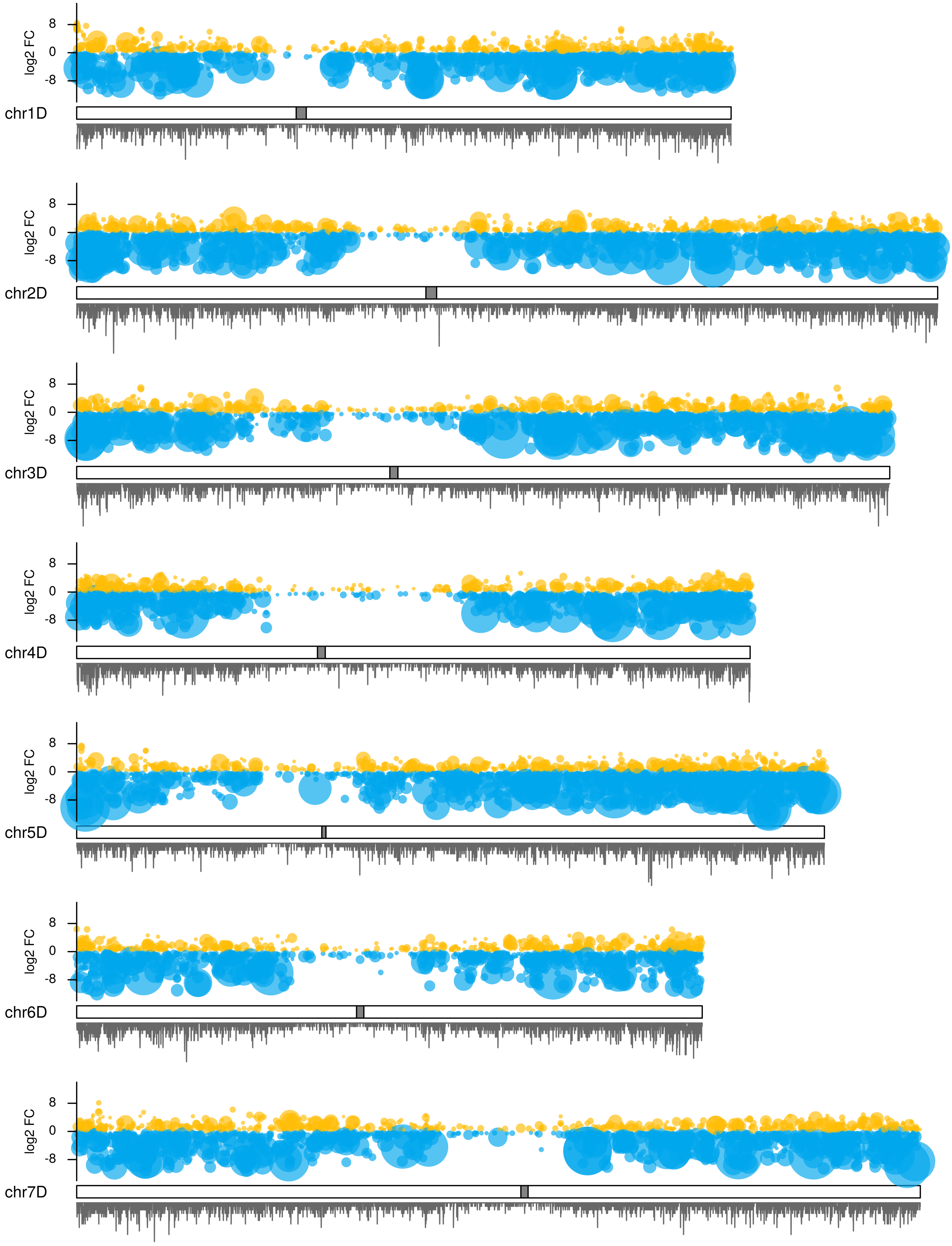

log2 FC
8
0
-8
chr1D
chr2D
chr3D
chr4D
chr5D
chr6D
chr7D

Additional file 15. Chromosome level view of differentially expressed rachis transcripts (above) and gene density (below) of the wheat genome component of the CS-7EL addition line in response to F. graminearum infection. Transcripts are represented as dots with color representing direction of change in expression, vertical position representing degree of difference in expression and dot size representing statistical significance.
