# Supplementary material for: Genomic sequencing of Thinopyrum elongatum chromosome arm 7EL, carrying fusarium head blight resistance, and characterization of its impact on the transcriptome of the introgressed line CS-7EL
Source: BMC Genomics. 2022 Mar 23;23:228. doi: 10.1186/s12864-022-08433-8 (PMC8944066; doi:10.1186/s12864-022-08433-8)
Supplement: Supplementary file 16 — Additional file 16. [file 12864_2022_8433_MOESM16_ESM.docx]

Additional file 16. Functionally annotated 7EL transcripts with significant differential expression between water and *F. graminearum* treatments in CS-7EL, grouped in functional categories.

| **Functional category** | Number of DE transcripts | |
| --- | --- | --- |
|  | Up by FG | Down by FG |
| LncRNA | 15 | 1 |
| Secondary metabolism | 11 | 2 |
| Signaling | 6 | 1 |
| Regulation of transcription | 6 | 4 |
| Disease resistance/stress response | 5 | 1 |
| Transport | 3 | 3 |
| Primary metabolism | 2 | 0 |
| Protein catabolism | 2 | 0 |
| Other functions | 1 | 0 |
| Uncharacterized/unannotated proteins | 150 | 23 |
