# Supplementary material for: Genomic sequencing of Thinopyrum elongatum chromosome arm 7EL, carrying fusarium head blight resistance, and characterization of its impact on the transcriptome of the introgressed line CS-7EL
Source: BMC Genomics. 2022 Mar 23;23:228. doi: 10.1186/s12864-022-08433-8 (PMC8944066; doi:10.1186/s12864-022-08433-8)
Supplement: Supplementary file 20 — Additional file 20. [file 12864_2022_8433_MOESM20_ESM.pdf]

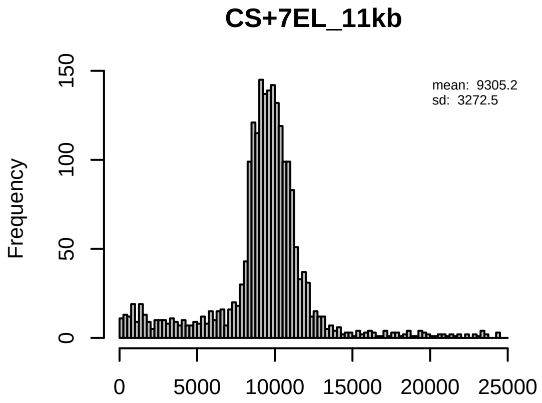
CS+7EL_11kb
mean: 9305.2
sd: 3272.5
Frequency
0
50
100
150
0
5000
10000
15000
20000
25000

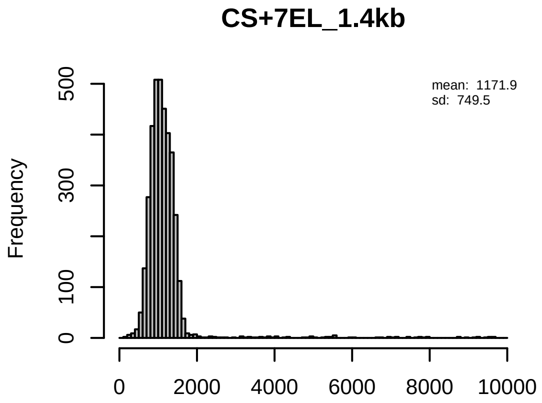
CS+7EL_1.4kb
mean: 1171.9
sd: 749.5
Frequency
0
100
300
500
0
2000
4000
6000
8000
10000

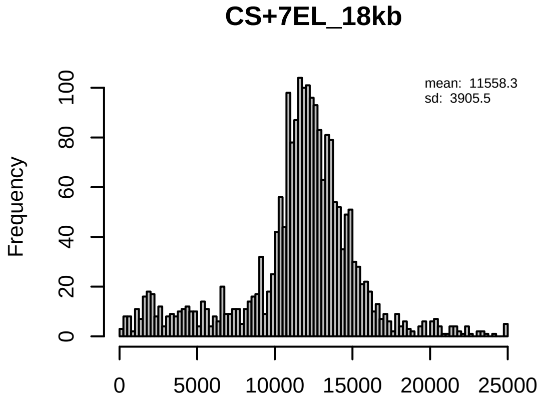
CS+7EL_18kb
mean: 11558.3
sd: 3905.5
Frequency
0
20
40
60
80
100
0
5000
10000
15000
20000
25000

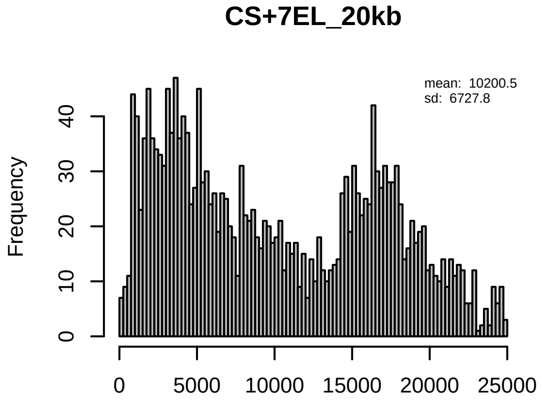
CS+7EL_20kb
mean: 10200.5
sd: 6727.8
Frequency
0
10
20
30
40
0
5000
10000
15000
20000
25000

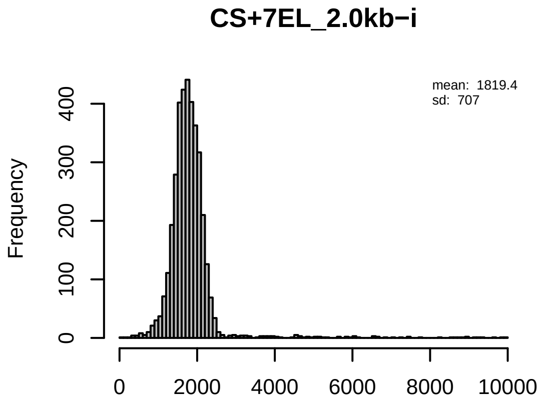
CS+7EL_2.0kb−i
mean: 1819.4
sd: 707
Frequency
0
100
200
300
400
0
2000
4000
6000
8000
10000

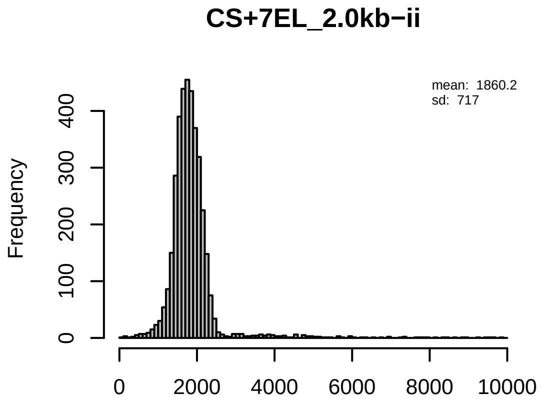
CS+7EL_2.0kb−ii
mean: 1860.2
sd: 717
Frequency
0
100
200
300
400
0
2000
4000
6000
8000
10000

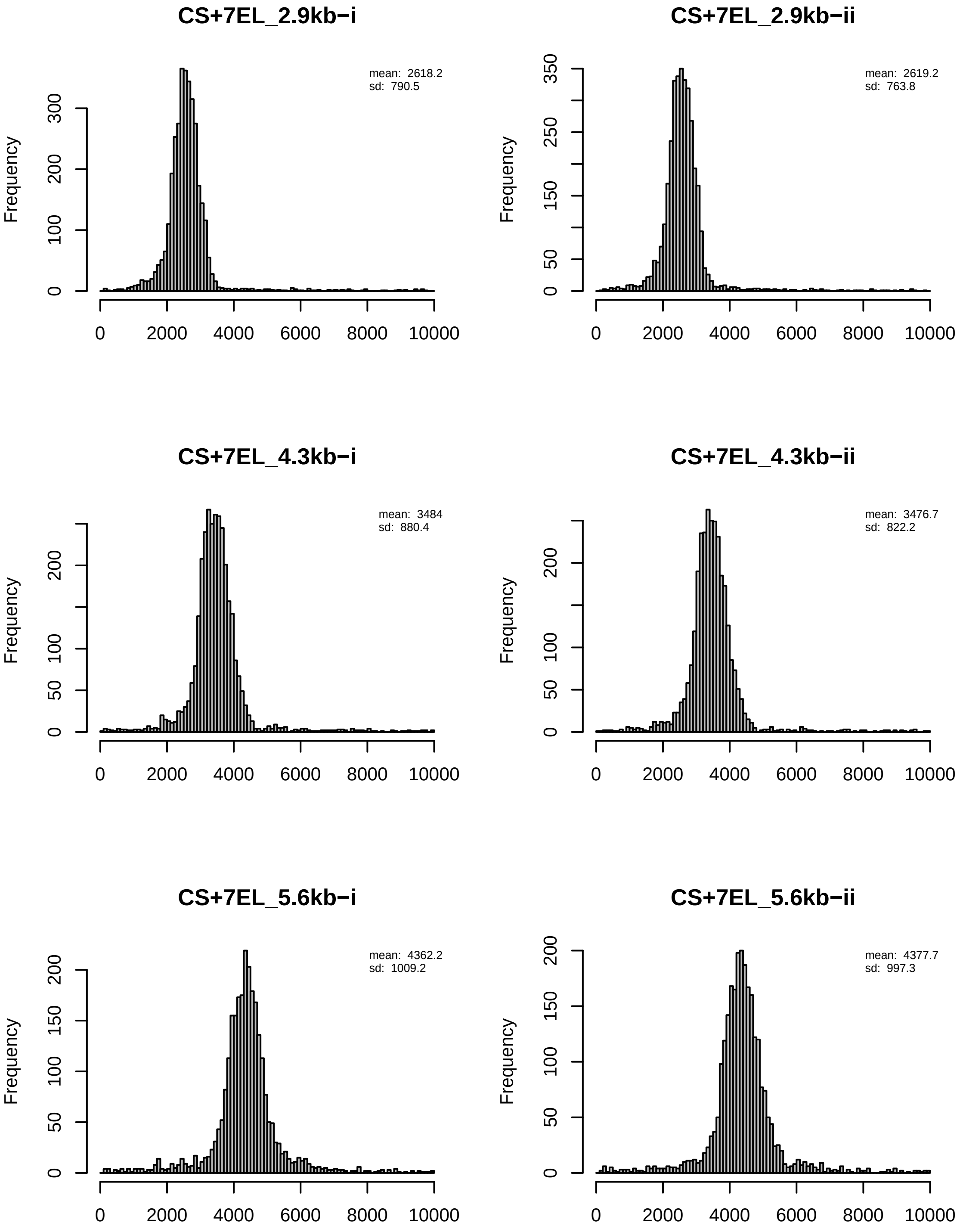
CS+7EL_2.9kb−i
mean: 2618.2
sd: 790.5
CS+7EL_2.9kb−ii
mean: 2619.2
sd: 763.8
CS+7EL_4.3kb−i
mean: 3484
sd: 880.4
CS+7EL_4.3kb−ii
mean: 3476.7
sd: 822.2
CS+7EL_5.6kb−i
mean: 4362.2
sd: 1009.2
CS+7EL_5.6kb−ii
mean: 4377.7
sd: 997.3
Frequency
0
2000
4000
6000
8000
10000

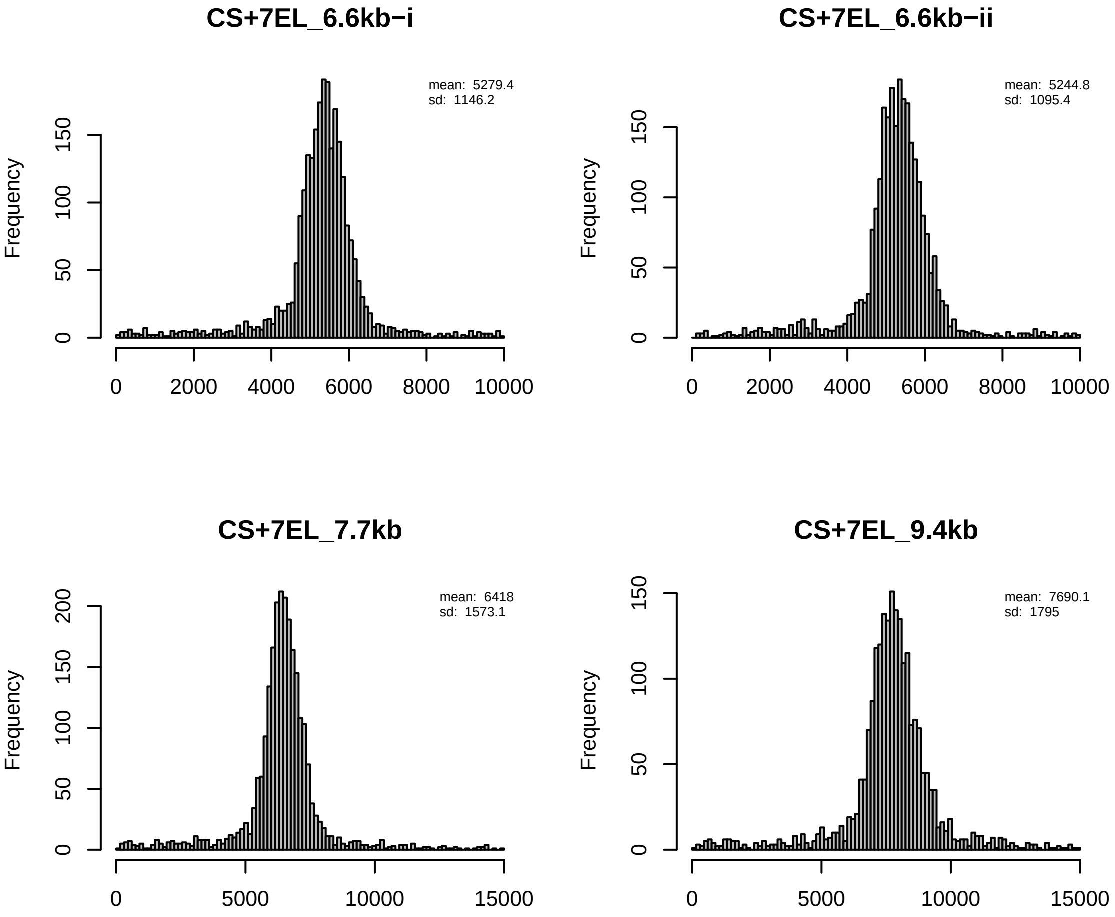

Additional file 20. Distributions of Nextera mate-pair mapping distances in reverse-forward (outward) orientationSuffix ".HQ" denotes mate pairs for which a junction sequence was identified during read processing. Suffix ".LQ" denotes mate pairs for which a junction sequence was not identified during read processing.
