# Supplementary material for: Genomic sequencing of Thinopyrum elongatum chromosome arm 7EL, carrying fusarium head blight resistance, and characterization of its impact on the transcriptome of the introgressed line CS-7EL
Source: BMC Genomics. 2022 Mar 23;23:228. doi: 10.1186/s12864-022-08433-8 (PMC8944066; doi:10.1186/s12864-022-08433-8)
Supplement: Supplementary file 21 — Additional file 21. [file 12864_2022_8433_MOESM21_ESM.pdf]

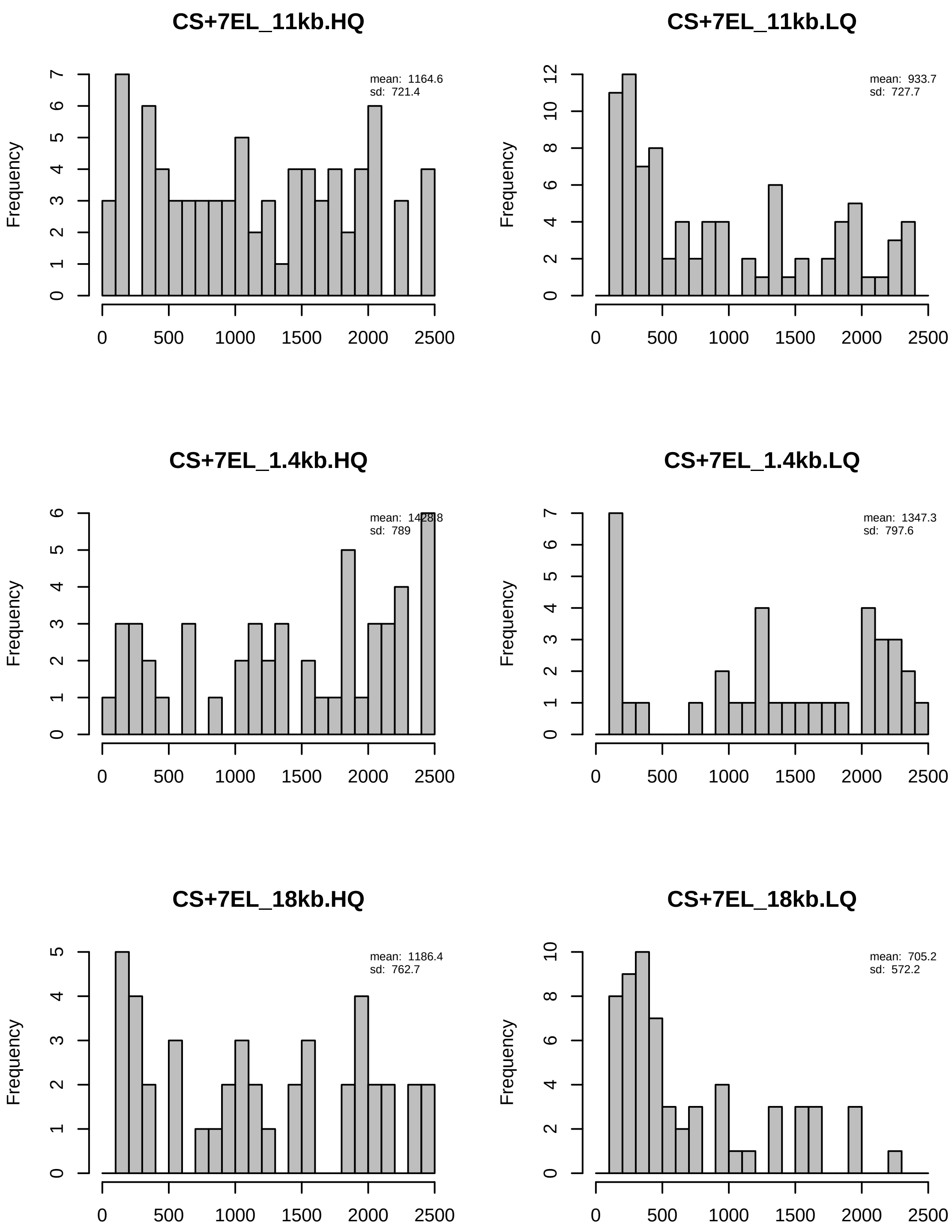
CS+7EL_11kb.HQ
mean: 1164.6
sd: 721.4
CS+7EL_11kb.LQ
mean: 933.7
sd: 727.7
CS+7EL_1.4kb.HQ
mean: 1428.8
sd: 789
CS+7EL_1.4kb.LQ
mean: 1347.3
sd: 797.6
CS+7EL_18kb.HQ
mean: 1186.4
sd: 762.7
CS+7EL_18kb.LQ
mean: 705.2
sd: 572.2
Frequency

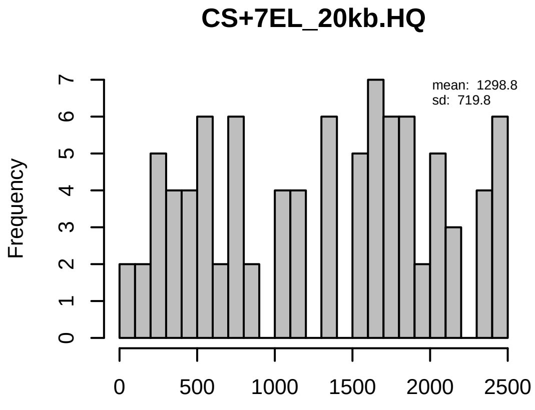
CS+7EL_20kb.HQ
mean: 1298.8
sd: 719.8
Frequency

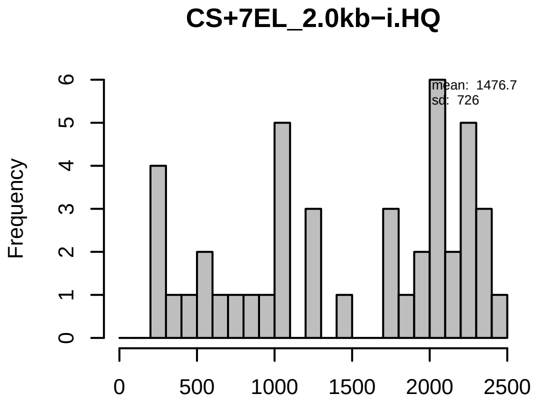
CS+7EL_2.0kb−i.HQ
mean: 1476.7
sd: 726
Frequency

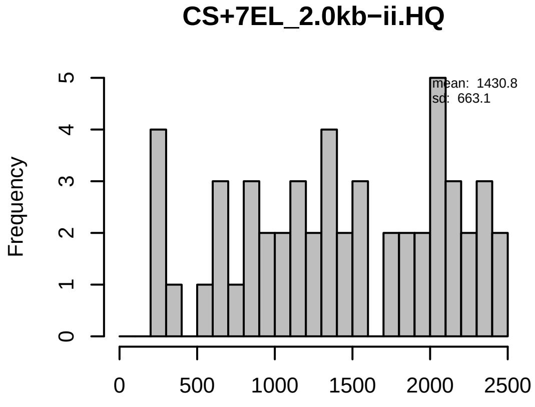
CS+7EL_2.0kb−ii.HQ
mean: 1430.8
sd: 663.1
Frequency

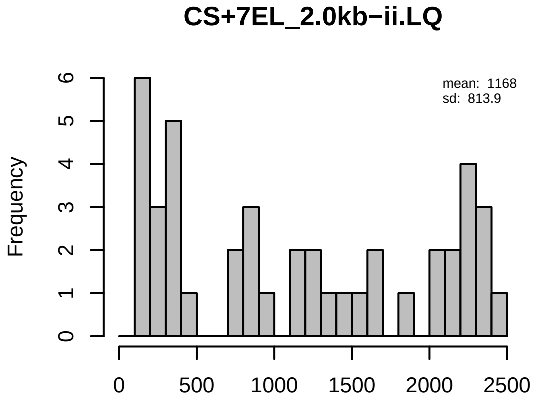
CS+7EL_2.0kb−ii.LQ
mean: 1168
sd: 813.9
Frequency

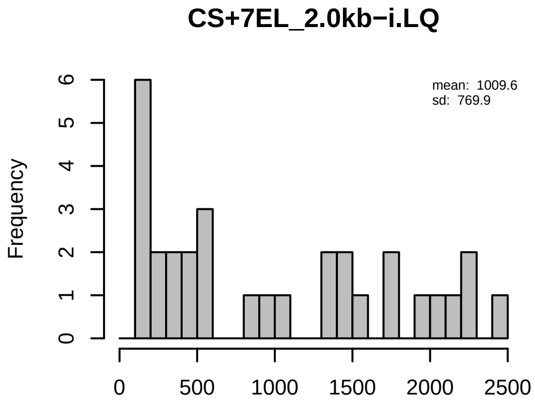
CS+7EL_2.0kb−i.LQ
mean: 1009.6
sd: 769.9
Frequency

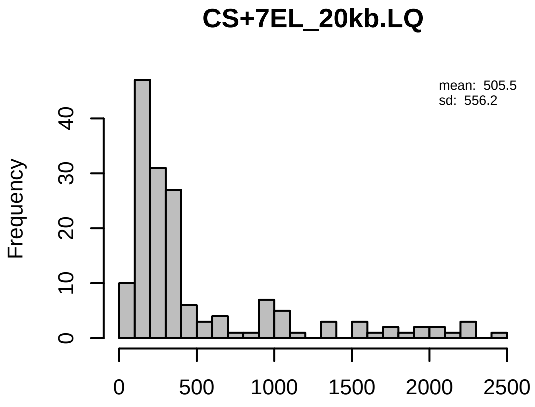
CS+7EL_20kb.LQ
mean: 505.5
sd: 556.2
Frequency

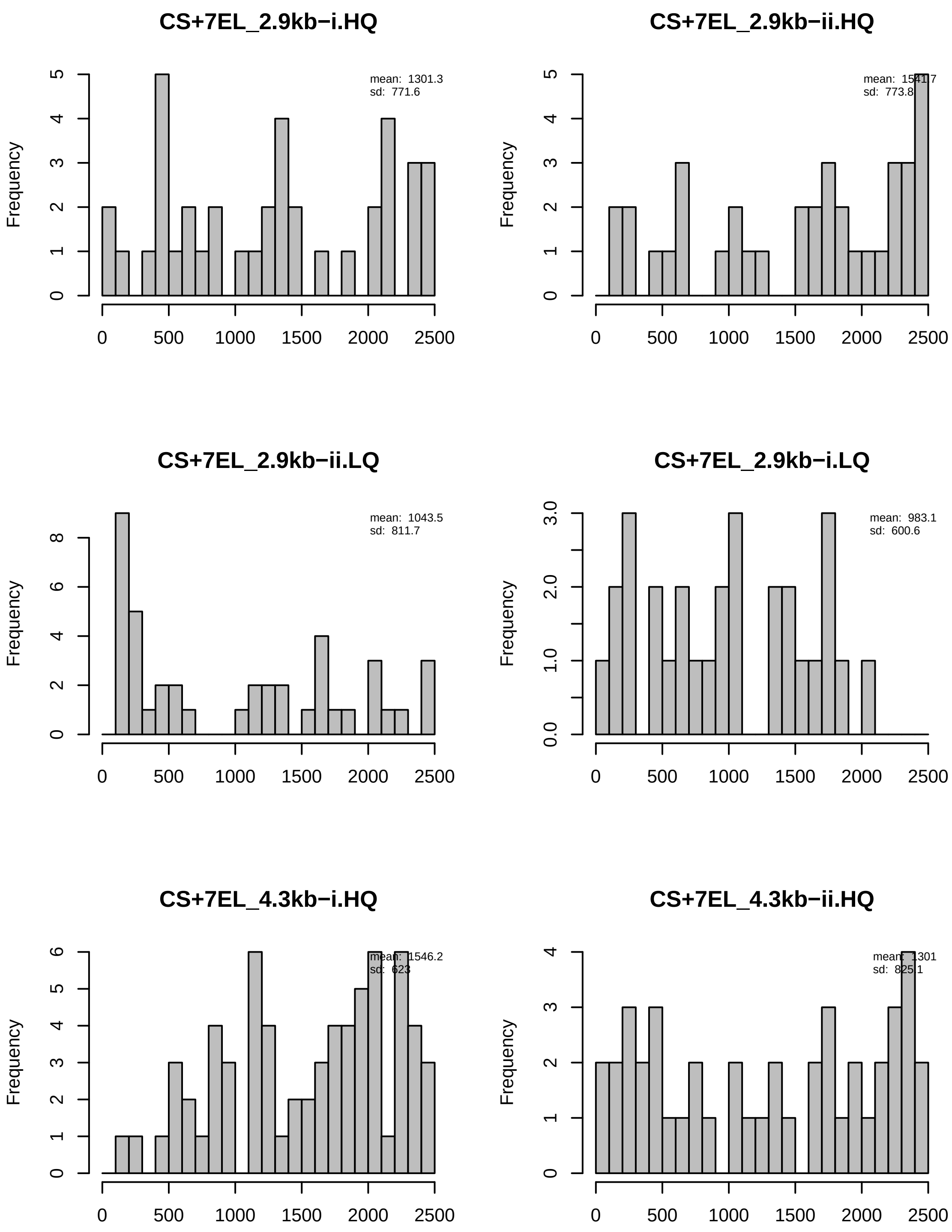
CS+7EL_2.9kb−i.HQ
mean: 1301.3
sd: 771.6
CS+7EL_2.9kb−ii.HQ
mean: 1541.7
sd: 773.8
CS+7EL_2.9kb−ii.LQ
mean: 1043.5
sd: 811.7
CS+7EL_2.9kb−i.LQ
mean: 983.1
sd: 600.6
CS+7EL_4.3kb−i.HQ
mean: 1546.2
sd: 623
CS+7EL_4.3kb−ii.HQ
mean: 1301
sd: 825.1
Frequency
0
500
1000
1500
2000
2500

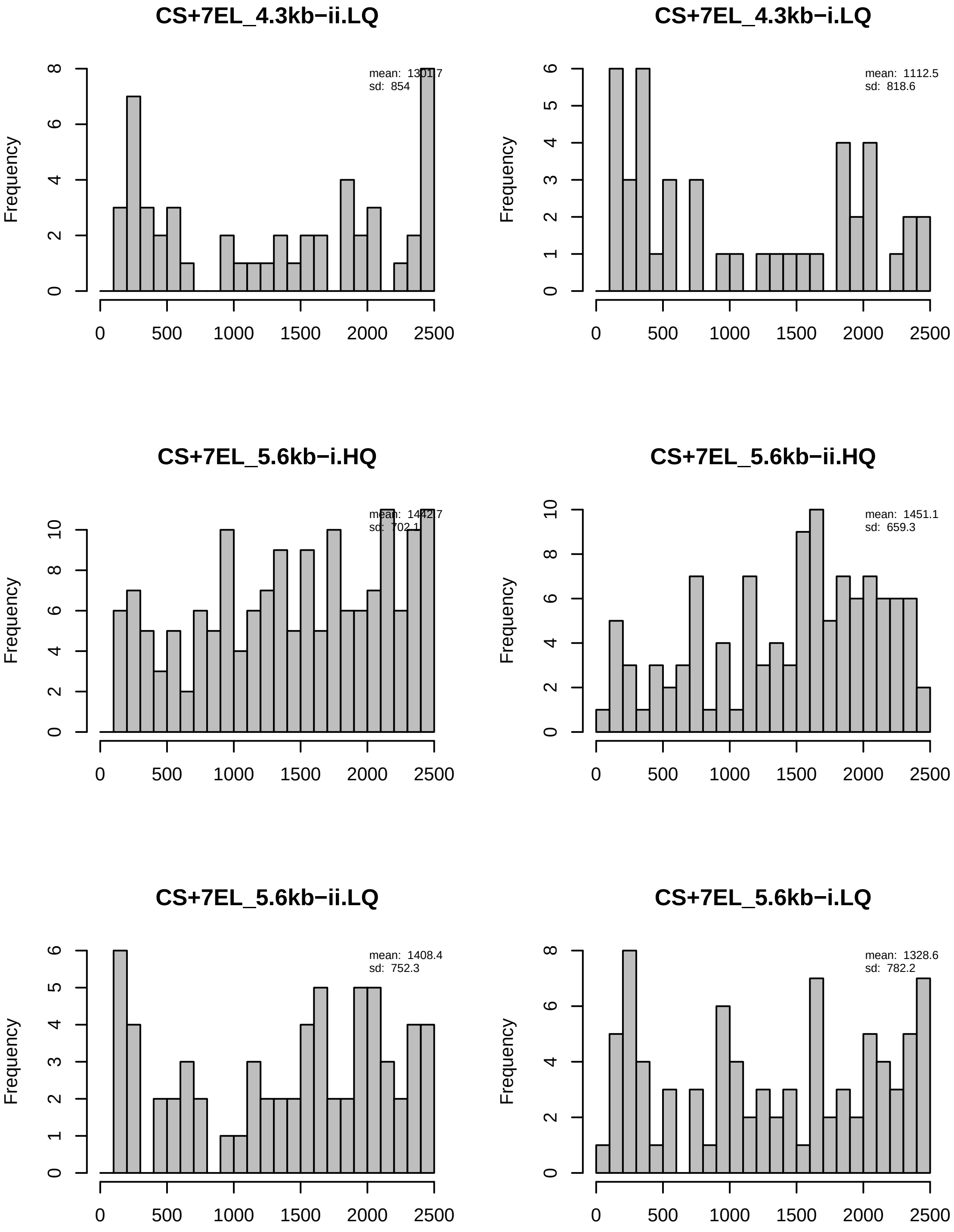
CS+7EL_4.3kb−ii.LQ
mean: 1301.7
sd: 854
CS+7EL_4.3kb−i.LQ
mean: 1112.5
sd: 818.6
CS+7EL_5.6kb−i.HQ
mean: 1442.7
sd: 702.1
CS+7EL_5.6kb−ii.HQ
mean: 1451.1
sd: 659.3
CS+7EL_5.6kb−ii.LQ
mean: 1408.4
sd: 752.3
CS+7EL_5.6kb−i.LQ
mean: 1328.6
sd: 782.2
Frequency
0
500
1000
1500
2000
2500

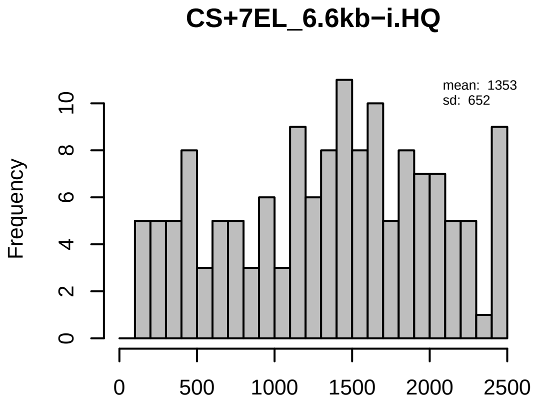
CS+7EL_6.6kb−i.HQ
mean: 1353
sd: 652
Frequency
0 2 4 6 8 10
0 500 1000 1500 2000 2500

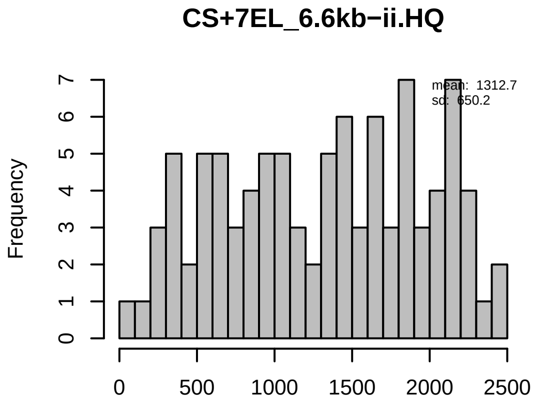
CS+7EL_6.6kb−ii.HQ
mean: 1312.7
sd: 650.2
Frequency
0 1 2 3 4 5 6 7
0 500 1000 1500 2000 2500

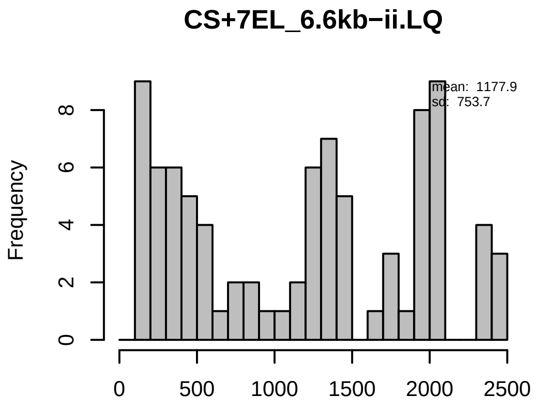
CS+7EL_6.6kb−ii.LQ
mean: 1177.9
sd: 753.7
Frequency
0 2 4 6 8
0 500 1000 1500 2000 2500

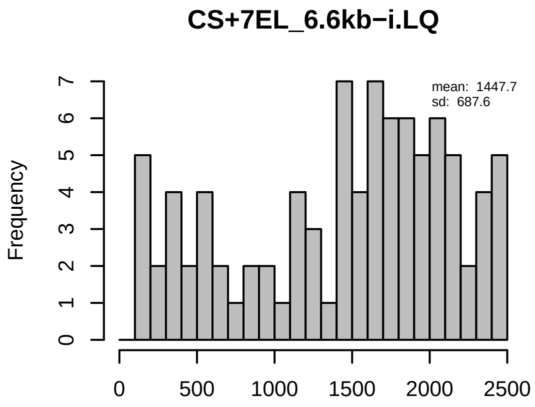
CS+7EL_6.6kb−i.LQ
mean: 1447.7
sd: 687.6
Frequency
0 1 2 3 4 5 6 7
0 500 1000 1500 2000 2500

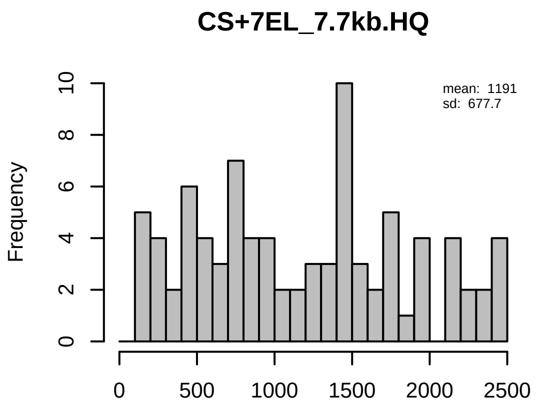
CS+7EL_7.7kb.HQ
mean: 1191
sd: 677.7
Frequency
0 2 4 6 8 10
0 500 1000 1500 2000 2500

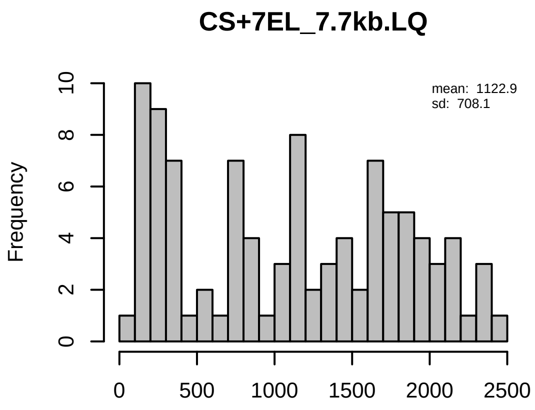
CS+7EL_7.7kb.LQ
mean: 1122.9
sd: 708.1
Frequency
0 2 4 6 8 10
0 500 1000 1500 2000 2500

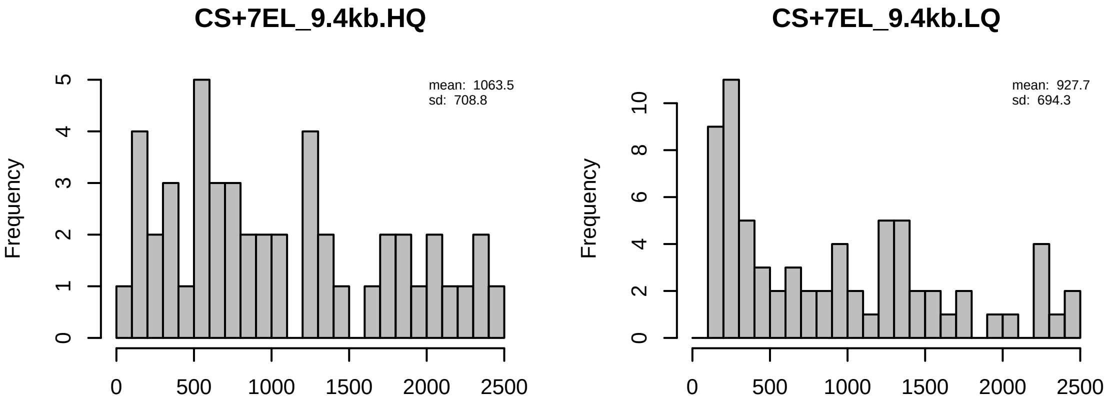

Additional file 21. Distributions of Nextera mate pairs that map in forward-reverse (inward) orientation
