# Supplementary material for: Genomic sequencing of Thinopyrum elongatum chromosome arm 7EL, carrying fusarium head blight resistance, and characterization of its impact on the transcriptome of the introgressed line CS-7EL
Source: BMC Genomics. 2022 Mar 23;23:228. doi: 10.1186/s12864-022-08433-8 (PMC8944066; doi:10.1186/s12864-022-08433-8)
Supplement: Supplementary file 22 — Additional file 22. [file 12864_2022_8433_MOESM22_ESM.docx]

Additional file 22. List of primers used for RT-qPCR analyses.

| **Transcript name** | **Functional annotation** | **Forward (5’ – 3’)** | **Reverse (5’ – 3’)** | **Source** |
| --- | --- | --- | --- | --- |
| MSTRG .145180 | WRKY79 | CCGTGTAACTGGATGTTAGCTCTC | CTTGTCGATGTAAGCCGAGTACAG | Additional files 11, 13, 14 |
| MSTRG .21806 | Glycosyltransferase | TACGTAGCACTTAGCAGGAC | GAACGGCATTCGATCTTTCC | Additional files 11, 13, 14 |
| MSTRG .60974 | Zinc finger protein ZAT | CATTGCGAGTCGAAGAACAG | CTTCAGGTTTCTTCTGAGCC | Additional files 11, 13, 14 |
| MSTRG .70787 | E3 ubiquitin-protein ligase | GTGTGTCGGTGTGTGTTTC | GTTTCGTGAGGACTTGCATAAT | Additional file 13 |
| FGSG_06257 | Glyceraldehyde 3-phosphate dehydrogenase  (FgGAPDH) | TGACTTGACTGTTCGCCTCGAGAA | ATGGAGGAGTTGGTGTTGCCGTTA | [50] |
| TraesCS2A01G246300 | Indole-3 acetaldehyde oxidase (TaAAOx) | CACAGCAGGATTTAAGCTCTGG | GGGATGGACTAATTTCACAGGC | [50] |
| TraesCS2A01G390200 | Heterogeneous nuclear ribonucleoprotein (Tahn-RNP-Q) | TCACCTTCGCCAAGCTCAGAACTA | AGTTGAACTTGCCCGAAAC | [50] |
| TraesCS7A01G313100 | Glyceraldehyde 3-phosphate dehydrogenase  (TaGAPDH) | AACTGTTCATGCCATCACTGCCAC | AGGACATACCAGTGAGCTTGCCAT | [59] |
